# Non-conventional bulk heterojunction nanoparticle photocatalysts for sacrificial hydrogen evolution from water†

**DOI:** 10.1039/d4ta03584d

**Published:** 2024-08-12

**Authors:** Jai-Ram Mistry, Ewan McQueen, Fabio Nudelman, Reiner Sebastian Sprick, Iain A. Wright

**Affiliations:** a Department of Chemistry, Loughborough University Epinal Way Loughborough Leicestershire LE11 3TU UK; b Department of Pure and Applied Chemistry, University of Strathclyde Thomas Graham Building, 295 Cathedral Street Glasgow G1 1XL UK sebastian.sprick@strath.ac.uk; c School of Chemistry, University of Edinburgh Joseph Black Building, David Brewster Road Edinburgh EH9 3FJ UK iain.wright@ed.ac.uk

## Abstract

Photocatalyst systems combining donor polymers with acceptor molecules have shown the highest evolution rates for sacrificial hydrogen production from water for organic systems to date. Here, new donor molecules have been designed and synthesised taking inspiration from the structure–performance relationships which have been established in the development of non-fullerene acceptors. While a conventional bulk heterojunction (BHJ) pairing consists of a donor polymer and acceptor small molecule, here we have successfully reversed this approach by using new p-type small molecules in combination with a n-type conjugated polymer to produce non-conventional BHJ (ncBHJ) nanoparticles. We have applied these ncBHJs as photocatalysts in the sacrificial hydrogen evolution from water, and the best performing heterojunction displayed high activity for sacrificial hydrogen production from water with a hydrogen evolution rate of 22 321 μmol h^−1^ g^−1^ which compares well with the state-of-the-art for conventional BHJ photocatalyst systems.

## Abstract 2

Bulk heterojunction nanoparticles composed of simple electron-donor molecules and an electron-acceptor polymer act as photocatalysts for the generation of hydrogen from water. Performance is dictated by the terminal groups of the donor molecules.

## Graphical abstract

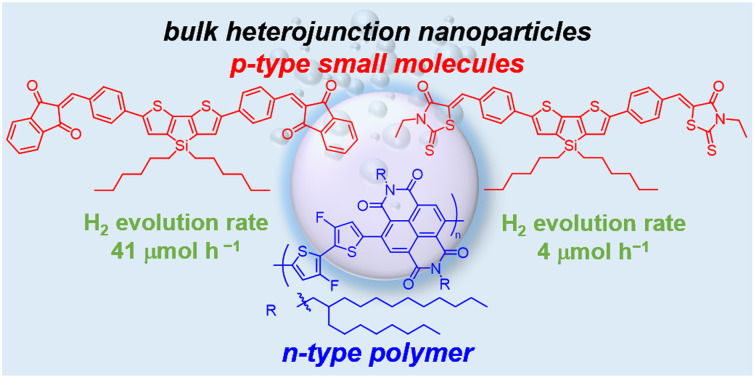

The production of hydrogen from water is an area of intense research due to the need for renewable fuels in the energy transition. For this, the production of hydrogen itself needs to occur cleanly and without emitting greenhouse gases; as such, photocatalytic water splitting has gained significant interest.^1^ Initially, a particular focus was on inorganic semiconductors as photocatalysts that drive water splitting suspended in aqueous solutions.^2^ However, over the last decade organic photocatalysts have emerged as attractive potential alternatives thanks to the control over properties including frontier orbital energy levels, charge transport and processing afforded by informed synthetic design, alongside the high efficiencies often demonstrated by these species.^3–9^ Including reports of overall water splitting.^10,11^

Informed by developments in bulk heterojunction (BHJ) organic solar cells (OSCs), the application of BHJ nanoparticles (NPs) has emerged as a promising approach in overcoming the high exciton binding energies in organic semiconductors thereby improving the efficiency of organic photocatalytic systems.^11^ Similar to OSCs an energy offset between a donor and acceptor component assists the charge separation of excitons into individual charges at the interface (outlined in Fig. 1a). The composition of a conventional BHJ is a p-type polymer donor in conjunction with an n-type small molecule acceptor. Historically the acceptors were fullerenes, but the high performances realised in contemporary BHJs has been driven by the emergence and rapid development of high-performance non-fullerene acceptors (NFAs).^11^

**Fig. 1:**
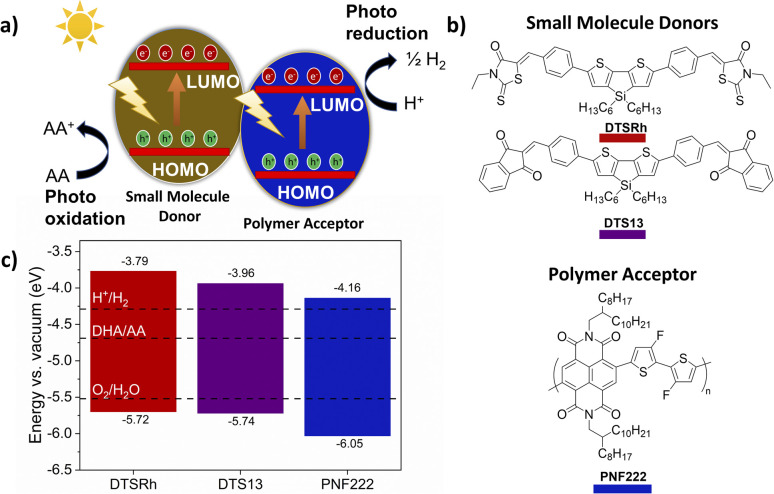
(a) Photocatalytic system based on two components; (b) structures of the small molecule donors and polymer acceptor used in this study; (c) energy levels of the small molecule donors and polymer acceptor determined from cyclic voltammetry measurements as outlined in the ESI.†

This conventional BHJ architecture has been retained as the general approach towards BHJs for photocatalytic water splitting, so donors and acceptors are typically selected from materials which have proven OSC efficiency.^12,13^ However, in OSCs the work function and interfaces of electrodes and the various layers in the devices must be considered and optimised for, but in photocatalytic water splitting the BHJ NPs are suspended in an aqueous solution and the reactions take place on the surface of the particles. Therefore, provided the thermodynamic demands of proton reduction and hole scavenger oxidation are met, hydrogen production might be observed on the metal co-catalysts such as palladium or platinum.^14,15^ The use of conventional BHJ NPs has resulted in a major absence of studies of n-type polymers for photocatalytic hydrogen generation. The potential of these materials is therefore largely unknown.

Here we present initial findings into non-conventional bulk heterojunction (ncBHJ) NPs which consist of an n-type polymer and p-type molecular donor, and demonstrate the viability of these ncBHJ NPs towards hydrogen production from water at ambient pressure. There were two immediate and interlinked challenges in exploring ncBHJ as photocatalysts, both of which arise from the aforementioned fact that BHJ OSCs have matured with a heavy focus on using electron-accepting small molecules in conjunction with donor polymers. There is a scarcity of benchmark n-type polymers in general and, concomitantly, there is also an absence of established donor molecules which show high performance in conjunction with such polymers.

The strategy adopted was to select one of the few commercially available n-type polymers which has energy level alignments appropriate for proton reduction and then synthesise donor molecules with complementary optoelectronic properties taking inspiration from NFA-design principles.

Naphthalene diimide polymer poly[[1,2,3,6,7,8-hexahydro-2,7-bis(2-octyldodecyl)-1,3,6,8-tetraoxobenzophenanthroline-4,9-diyl](3,3′-difluoro[2,2′-bithiophene]-5,5′-diyl)] PNF222 (Fig. 1b) was selected as the n-type polymer due to its moderately narrow *E*_g_ = 1.89 eV and its ionisation potential (IP) and electron affinity (EA) straddling the required potential window for water splitting and oxidation of sacrificial hole scavengers such as ascorbic acid (AA) (Fig. 1c) alongside its previous good performance in BHJ^16–20^ and other OSC devices.^21,22^ To ensure that the energetic requirements for water splitting were met, donor molecules with similarly wide *E*_g_ and IP/EA positively offset from those of the polymer are required.

Successes of fused-ring NFAs such as ITIC and Y6 (Fig. S1†) arise from a combination of their low and tuneable HOMO–LUMO gap (*E*_g_) alongside their tendency to interact well within themselves and with donor polymers to reduce losses.^23–28^ Structure–property relationships in NFAs are highly complex, but we considered that there are no clear reasons why structural motifs that have proven successful in NFAs should not lead to similarly effective donors for use alongside polymers of respectively greater n-type character. Both ITIC and Y6 have an overall acceptor–donor–acceptor structure with extended ring-fused cores which destabilise the HOMO level (decreasing IP), and terminal pendant acceptor units which stabilise the LUMO (decreasing EA). The ring-fused sp^2^-backbone in NFAs allows for significant overlap between the HOMO and LUMO resulting in very narrow *E*_g_. Considering these criteria, we designed and synthesised two new donors DTSRh and DTS13 (Fig. 1b). These molecules have terminal acceptors of more modest strength than those encountered in typical NFAs, namely *N*-ethylrhodanine and 1,3-indanedione respectively, and ring-fusion was limited to a central 4,4′-bis(*n*-hexyl)dithieno[3,2-*b*:2′,3′-*d*]silole (DTS) heterocycle which was also separated from the acceptors using phenyl π-spacers to produce quadrupolar acceptor–π–donor–π–acceptor type systems (for synthetic details see ESI, Scheme S1†). DTSRh and DTS13 are very simple molecules in contrast to the challenging synthesis typically associated with NFAs and their synthesis avoids the use of problematic organotin reagents and halogenated acceptor units.^29^

Cyclic voltammetry (Fig. S2 and S4†) was employed to obtain the IP and EA for each component, revealing that the thermodynamic requirements for water splitting have been met. The IP and EA of both DTS molecules straddle the proton reduction and AA oxidation potentials, and the required downhill energy offset between the donors and the polymer had been achieved. The IP of DTSRh and DTS13 were similar at −5.72 and −5.74 eV (*vs.* vacuum), respectively (Fig. 1c). The EA of DTS13 was determined to be at −3.96 eV, which is 0.17 eV below that of DTSRh (−3.79 eV) and offset by *ca.* +0.20 eV from that of PNF222 which was determined as −4.16 eV. Frontier orbital energies for DTSRh and DTS13 obtained from DFT (B3LYP/def2SVP) showed good general agreement with the values obtained experimentally (Tables S1, S2, Fig. S6 and S7†). ncBHJ NPs of DTSRh/PNF222 and DTS13/PNF222 in different weight ratios were fabricated (3 : 1, 1 : 1, 1 : 3, for the donor and acceptor respectively), through a mini emulsion process using sodium *n*-dodecyl sulfate (SDS) as the surfactant.^13^

UV/Vis spectroscopy (Fig. 2a and c) showed that the NPs have desirable broad absorption in the visible spectrum with absorbance bands up to *ca.* 825 nm. The long wavelength intramolecular charge transfer (CT) bands of the donors decrease in intensity as new band emerges at 548 nm for DTSRh and 589 nm for DTS13 indicating that intermolecular CT is occurring between the components in the NPs. The energy difference between these bands (0.16 eV) correlates very well with the difference in EA between the compounds. These changes were not observed in the spectra of solution phase mixtures of the discrete donor and acceptor nanoparticles, both of which showed clear isosbestic points (Fig. S5†) indicating that a ncBHJ blend in NP form is required for CT to take place.

**Fig. 2:**
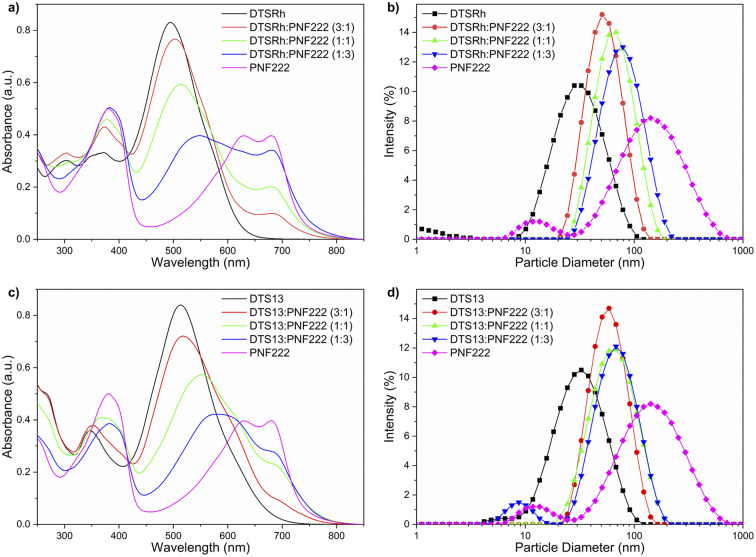
UV/Vis spectroscopy (left) and DLS responses (right) of NPs consisting of different ratios of PNF222 with: (a) and (b) DTSRh and (c) and (d) DTS13.

Unimodal size distributions and a relatively constant hydrodynamic diameter (*Z*_avg_) between each NP batch was confirmed by dynamic light scattering (DLS, Fig. 2b, d and Table S3†). The size of NPs directly impacts the total available surface area at the interface where the redox processes take place and thus influences the efficiency directly.^30^ Single-component NPs of DTSRh and DTS13 were on average smaller than those of PNF222 which is attributed to tighter packing and reduced free volume of the small molecules compared to the polymer. This was also observed in the ncBHJ NPs where those containing a larger proportion of PNF222 had a larger average particle diameter.

The photocatalytic activity of the ncBHJ NPs was determined using AA as the sacrificial hole scavenger and platinum acting as the co-catalyst under visible light irradiation (300 W Xe light source, AM 1.5 G filter) and atmospheric pressure. Photocatalytic experiments showed different activities depending on their composition (Fig. 3a): DTS13 : PNF222 in a 3 : 1 ratio showed the highest photocatalytic activity with a hydrogen evolution rate (HER) of 41 μmol h^−1^ measured over 5 hours. When directly comparing the 1 : 1 blends, DTS13 : PNF222 shows a much higher activity with HER of 29 μmol h^−1^ compared to 4 μmol h^−1^ for DTSRh : PNF222 over the course of 6 hours of irradiation.

**Fig. 3:**
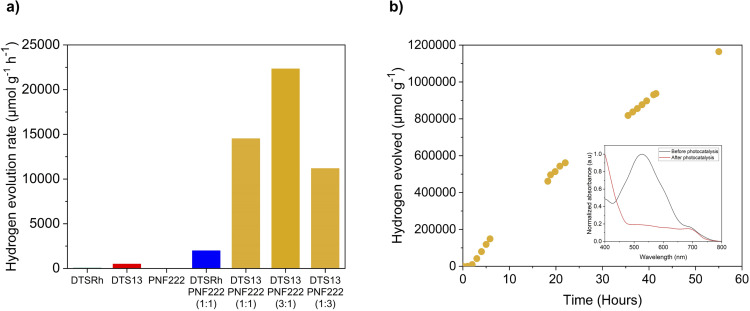
Photocatalytic hydrogen evolution data. (a) Mass normalised hydrogen evolution rates for the DTS13 : PNF222 ncBHJ nanoparticles with different blend ratios of donor and acceptor, DTSRh : PNF222 ncBHJ nanoparticles in a 1 : 1 blend and the single component nanoparticles of DTS13, DTSRh and PNF222, respectively. (b) Mass normalised hydrogen evolution rate of DTS13 : PNF222 (3 : 1) ncBHJ nanoparticles over 55 hours of photoirradiation. Experimental conditions: 4 mL of 0.5 mg mL^−1^ nanoparticle dispersion to obtain 2 mg of nanoparticles, 10 wt% Pt (1.25 mL from a 0.4 mg mL^−1^ aqueous K_2_PtCl_6_ solution), 19.75 mL of a 0.2 M ascorbic acid solution added to make the total volume up to 25 mL, degassed by nitrogen bubbling, irradiated with a 300 W Xe light source equipped with an AM 1.5 G filter. The graph inset displays the normalised UV/Vis absorption spectra of DTS13 : PNF222 (3 : 1) ncBHJ nanoparticles before and after 55 hours of photoirradiation.

Cryo-transmission electron microscopy (CryoTEM) of the 1 : 1 blends (Fig. 4) revealed that the NPs containing DTS13 were well-defined while those from DTSRh were agglomerates which may explain their relative performances. No fine structure was observed in the NPs of either blend. Control experiments showed that the DTS13 : PNF222 1 : 1 ncBHJ NPs are inactive for photocatalytic hydrogen evolution in the dark, without the hole scavenger present, and without the platinum co-catalyst (Fig. S9†).

**Fig. 4:**
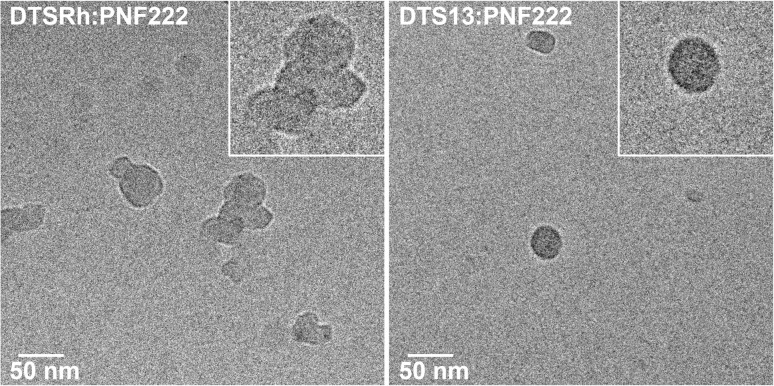
Cryo-transmission electron microscopy images of DTSRh : PNF222 (left) and DTS13 : PNF222 (right). Insets show higher magnification of the particles.

Cycle stability of the photocatalytic system was tested for the DTS13 : PNF222 1 : 1 ncBHJ NPs (Fig. S10†). When initially left in the dark for 2 hours no hydrogen evolution was observed. Following this the sample was irradiated for 5 hours and a hydrogen evolution rate of 12 807 μmol g^−1^ h^−1^ was determined. The sample was then degassed and irradiated for a further 9 hours (2nd run) showing a HER of 5954 μmol g^−1^ h^−1^. The addition of AA resulted in a very similar photocatalytic activity in the 3rd run compared to the 2nd run with a HER of 6833 μmol g^−1^ h^−1^. This shows that the AA concentration is not limiting the photocatalytic activity over time and that the system is relatively durable for photocatalytic hydrogen evolution, despite a small reduction in activity compared to the first run.

As shown in (Fig. 3a), the system incorporating a 3 : 1 blend of DTS13 and PNF222 displayed the greatest activity for sacrificial hydrogen production over 5 hours, thus we studied the stability of these ncBHJ NPs over an even larger irradiation timescale (Fig. 3b). Over 55 hours, hydrogen evolution persists with a very similar HER to that of the 5 hours experiment (22 321 μmol g^−1^ h^−1^ over 5 hours *vs.* 22 425 μmol g^−1^ h^−1^ over 55 hours), highlighting the high durability of this particular photocatalyst for hydrogen production. We can observe that the HER gradually does begin to slow at longer timescales, so to gain insight into possible deactivation pathways that are arising over time we investigated the absorption properties before and after photocatalysis. A clear depletion of the main absorption band in the UV/Vis spectrum associated with both the intramolecular CT of DTS13 and the intermolecular CT with the polymer was observed for the DTS13 : PNF222 3 : 1 blend after photoirradiation compared to before photoirradiation (see absorption profile inset in Fig. 3b). The band is not entirely depleted after this time and the catalyst remains active, but this likely indicates some evolution of charge distribution within and/or on the surface of the particles, or a change in the structure of the heterojunction or DTS13 itself as potential reasons for the slow drop off in activity over time.

We also investigated if aggregation was a potential deactivation pathway contributing to decreased activity over time by measuring particle size distributions after photocatalysis using the DTS13 : PNF222 1 : 1, which showed no distinctive increase in particle size distribution (*Z*_avg_ = 91 nm before and 72 nm after photoirradiation in the presence of AA, Fig. S8 and Table S4†) thus inferring aggregation is not observed in these systems. Interestingly, the *Z*_avg_ for DTS13 : PNF222 1 : 1 was measured to be 50 nm in the stock solution, suggesting predetermined aggregation occurs when the medium has the scavenging reagents used in this work.

Triethylamine (TEA) was also investigated as a hole scavenger as it has been shown to be active in photocatalytic hydrogen production systems with organic semiconductors,^31^ however, only limited activity was observed when used with the DTS13 : PNF222 1 : 1 system (≤0.6 μmol of hydrogen after 3 hours of photoirradiation, Fig. S9†). We observe somewhat similar changes to the absorption profile of the nanoparticles after photoirradiation when compared to the long timescale experiment in Fig. 3b, with the band at 514 nm in the UV/Vis spectrum, being depleted (Fig. S11†) which implies that similar changes in the heterojunction are occurring. A slower depletion also occurs in the presence of AA which suggest that the presence of TEA is accelerating heterojunction degradation pathways.

As expected, single component NPs overall demonstrated little activity. DTS13 displayed a HER of 1 μmol h^−1^ after 6 hours (Fig. S12†) which is an order of magnitude greater than that observed for DTSRh. While the frontier orbital distributions are similar, as is the overall profile and intensity of the CT band in their UV/Vis spectra, DTS13 has a lower EA and thereby narrower *E*_g_ than DTSRh. Single component photocatalysts are often hampered by the challenging requirements of having a sufficiently narrow *E*_g_ to facilitate photoactivation alongside frontier orbital energies which will provide high enough overpotentials to drive the reaction. While morphology differences may also be a factor, it appears that DTS13 is sitting closer to this energetic “sweet-spot” than DTSRh thereby allowing it to produce measurable amounts of hydrogen.

## Conclusions

The conventional polymer donor : small molecule acceptor BHJ composition has been successfully inverted. Two new NFA-inspired donor materials DTSRh and DTS13 have been designed and synthesised, and ncBHJ NPs of DTS13 in combination with the n-type polymer PNF222 display photocatalytic HER of up to 22 321 μmol h^−1^ g^−1^. The new donors are cost effective and simple to synthesise, in contrast with state-of-the-art NFAs. While the overall rates obtained from this initial study are lower than literature examples of conventional BHJ NPs (Table S5†) this work stands in contrast with many of these by using a novel BHJ configuration. Furthermore, the results presented have been obtained with minimal optimisation. We have used modest Pt loading and evaluated the ncBHJ NPs at ambient pressures and without tuning of the pH of the photocatalysis mixture. By screening further surfactant and polymer combinations it can be anticipated that even more active ncBHJ NPs will be realised. This approach has proven simple to apply and appears to be readily adaptable to existing materials, while also providing impetus for renewed development of n-type conducting polymers.

## Data availability

Data supporting this article has been included as part of the ESI.†

## Conflicts of interest

There are no conflicts to declare.

## Supplementary Material

TA-012-D4TA03584D-s001
