# Supplementary material for: Non-conventional bulk heterojunction nanoparticle photocatalysts for sacrificial hydrogen evolution from water
Source: J Mater Chem A Mater. 2024 Aug 12;12(35):23411–5. doi: 10.1039/d4ta03584d (PMC11352093; doi:10.1039/d4ta03584d)
Supplement: TA-012-D4TA03584D-s001 [file TA-012-D4TA03584D-s001.pdf]

This journal is © The Royal Society of Chemistry 2024

Supplementary Information

for

Non-Conventional Bulk Heterojunction Nanoparticle Photocatalysts for Sacrificial Hydrogen Evolution from Water

Jai-Ram Mistry,[a] Ewan McQueen,[b] Fabio Nudelman,[c] Reiner Sebastian Sprick,[b*] and Iain A. Wright[c*]

[a] Department of Chemistry, School of Science, Loughborough University, Loughborough LE11 3TU, United Kingdom.

[b] Department of Pure and Applied Chemistry, University of Strathclyde, Thomas Graham Building, 295 Cathedral Street, Glasgow G1 1XL, United Kingdom. Email: sebastian.sprick@strath.ac.uk

[c] School of Chemistry, University of Edinburgh, Joseph Black Building, Edinburgh EH9 3FJ, United Kingdom. Email: iain.wright@ed.ac.uk

Contents

**General Experimental**	**S3**
**Structures of Y6 and ITIC**	**S6**
**Synthetic Methods**	**S7**
**^{1}H and ^{13}C NMR Spectra**	**S15**
**Molecular weight and Optoelectronic Properties of PNF222**	**S18**
**Optoelectronic Properties of DTSRh and DTS13**	**S19**
**Computational Results**	**S20**
**Dynamic Light Scattering Results**	**S26**
**Additional Photocatalysis Figures**	**S28**
**References**	**S32**

General Experimental

Synthesis and characterisation. All reactants and reagents were purchased from commercial suppliers and used without further purification unless otherwise stated. *N*-Bromosuccinimide was recrystallised from water prior to use[S1] **PNF222** was purchased from Ossila (batch M2052A2). All reactions were performed under an inert atmosphere in oven dried glassware unless otherwise stated.

Column chromatography was carried out using silica gel (60, 40-60 um mesh Fluorochem). Analytical thin-layer chromatography was performed on precoated aluminium backed silica gel 60 F254 plates (Merck) and Macherey-Nagel ALUGRAM Alox N/UV254 aluminium backed plates (Fisher), which were approximately 2.5 cm × 5 cm in size and visualized using ultraviolet light (254/365 nm).

NMR spectra were recorded on Jeol ECS 400 MHz and Jeol ECZ 500 MHz spectrometers. Chemical shifts are reported in ppm downfield of tetramethylsilane (TMS) using TMS or the residual solvent as an internal reference. NMR spectra were processed using MestReNova. Multiplicities are reported as singlet (s), doublet (d), triplet (t), quartet (q) and multiplet (m).

IR spectra were collected on a Thermo Scientific Nicolet FTIR spectrometer.

High resolution mass spectrometry (HRMS) was measured on a Thermo-Finnigan LTQ FT mass spectrometer. Samples were prepared in MeCN or a 9:1 MeCN:CH_2Cl_2 mixture.

UV-vis absorbance spectra were measured using a UV-1800 spectrophotometer (Shimadzu) and UVProbe version 2.33 software.

Emission spectra were recorded on a Horiba Fluoromax® 4 luminescence spectrometer using FluorEssence™ software. Photo-luminescent quantum yields (PLQY) measurements were carried out relative to that of Rhodamine-6G (0.94 in ethanol[S2]).

Elemental analyses were obtained on an Exeter Analytical CE440 Elemental Analyser.

Melting points were determined in open-ended capillaries using a Stuart Scientific SMP10 melting point apparatus at a ramping rate of 1 °C/min. They are recorded to the nearest 1 °C and are uncorrected.

TGA were performed using a TA ST Q600 instrument. Two alumina crucibles with a small amount of sample (1−5 mg) and an equal (±0.01 mg) amount of alumina reference powder were used for the TGA and the temperature was increased at a rate of 10 °C/min from 25 °C to 1000 °C. Data obtained was analysed using TA Instruments Universal Analysis 2000 (Version 4.5A, Build 4.5.0.5) software.

Cyclic voltammetry was recorded in CH_2Cl_2 solution using a Autolab Potentiostat (PGSTAT128) and NOVA 2.1 software with internal resistance compensation applied. A glassy carbon disk working electrode, Pt wire, and Ag/Ag^+ (0.1 M $AgNO_3$ in acetonitrile) were used as the working, counter, and reference electrodes, respectively. An analyte molarity of ca. 10^{-3} M was employed in the presence of 10^{-1} M ($TBAPF_6$) as a supporting electrolyte. Solutions were degassed with Ar and experiments run under a blanket of Ar. The working electrode was polished between each experiment. Measurements were corrected to the half-wave potential of the ferrocene/ferrocenium (Fc/Fc^+) redox couple as an internal standard. Ionisation potential (IP) and electron affinity (EA) values were approximated using the voltammetry results and are presented on the absolute electrochemical scale (0 V vs. standard hydrogen electrode (SHE) = −4.44 V vs. vacuum).[S3] The IP and EA were obtained by considering both the oxidation potential of Fc/Fc^+ in CH_2Cl_2 (+0.46 V vs. SCE)[S4] and the fact that SCE has a potential of +0.24 V vs. SHE.[S5] The oxidation potential of Fc/Fc^+ can therefore be estimated as +0.70 V vs. SHE. According to the Nernst equation, a further adjustment of +0.12 V is required due to the catalysis occurring in a pH 2 solution. The IP and EA were then approximated using the onset of the oxidation (E_{onset}^{ox}) or reduction (E_{onset}^{red}) wave respectively from the cyclic voltammetry according to the formulas:

$$IP = -4.44 - (+0.70) - (+0.12) - E_{onset}^{ox}$$

$$EA = -4.44 - (+0.70) - (+0.12) - E_{onset}^{red}$$

Dynamic light scattering (DLS) measurements were carried out on a Malvern Zetasizer Nano. Nanoparticle dispersions were diluted by placing 50 µL of 0.5 mg/mL dispersion in 3 mL of DI water (pre filtered through 0.45 µ PTFE) prior to measurements. Measurements were performed in triplicate and averaged.

Density functional theory calculations were performed with Orca v 5 [S6] using the B3LYP hybrid functional[S7] and def2-SVP basis set.[S8,S9]

Preparation of photocatalyst nanoparticles. To prepare the nanoparticles, individual stock solutions of **DTSRh**, **DTS13** and **PNF222** in chloroform solvent at a concentration of 0.50 mg mL^{-1} were prepared. These solutions were heated overnight at 80 °C to ensure complete dissolution then allowed to cool before being filtered using a 0.45-µm PTFE filter. The nanoparticle precursor solutions were created by mixing the stock solutions in the desired nanoparticle composition ratio with total volume of 10 mL. Subsequently, the nanoparticle precursor solution was added to a pre-prepared 0.5 wt. % solution of sodium dodecyl sulfate (SDS) surfactant in deionised water (10 mL) resulting in a bilayer. This mixture was vigorously stirred for 15 min at 40 °C to create a pre-emulsion, which was further subjected to sonication

for 20 min using an ultrasonic processor (Misonix Sonicator Ultrasonic Processor XL fitted with a 3 mm microtip) to form a mini emulsion. The mini-emulsion was heated at 85 °C under a stream of nitrogen with gentle stirring to remove the chloroform, leaving a surfactant-stabilised nanoparticle dispersion in water. Finally, the dispersion was filtered (0.45-μm PTFE) to remove any large aggregates before being stored in an amber vial to avoid over exposure to light.

Hydrogen evolution measurements. Hydrogen evolution from **DTSRh**, **DTS13** and **PNF222** nanoparticles was measured using ascorbic acid as the sacrificial hole scavenger and platinum as the co-catalyst. **DTSRh**, **DTS13** and **PNF222** nanoparticles with varying ratios of each component (2 mg from a 0.5 mg mL^{-1} dispersions) loaded with 10% platinum (1.25 mL from a 0.4 mg mL^{-1} aqueous potassium hexachloroplatinate solution) were made up to 25 mL total volume using an ascorbic acid solution (0.2 M) in a quartz flask with stirrer. The flask was degassed with nitrogen and irradiated with a 300 W Xe Oriel light source fitted with am AM1.5G filter. The hydrogen evolution rates were determined by taking gas from the head space at regular intervals and running these on a gas chromatograph (Thermo Scientific Trace 1300) equipped with a molecular sieve column. The amounts of hydrogen being produced were then calculated by using an external calibration.

Cryo-transmission electron microscopy (CryoTEM). R2/2 Quantifoil cryoTEM Au grids (Quantifoil Micro Tools GmbH) were plasma-treated for 1 minute using a Pelco Glow Discharge system. Solutions of **DTSRh**:**PNF222** (1:1) and **DTS13**:**PNF222** (1:1) were prepared as described above and were added in aliquots of 3 μl to the grid then plunge-frozen in liquid ethane using a vitrification robot (FEI Vitrobot Mark IV), at 21 °C and 100% humidity. CryoTEM imaging was done on a FEI Tecnai F20 transmission electron microscope operating at 200 kV, equipped with a Gatan cryoholder at −170 °C. Images were recorded using a Gatan CMOS Rio camera.

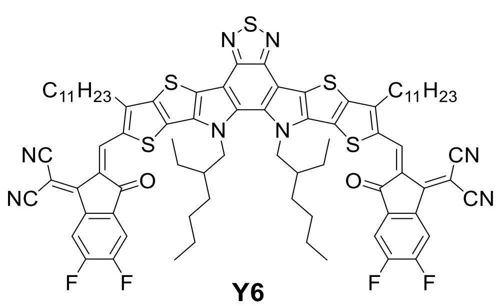

Y6

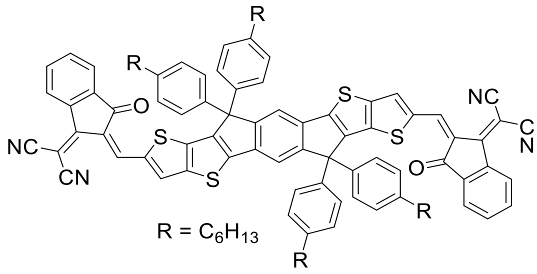

ITIC

Figure S1: Molecular structures of **Y6** and **ITIC**

Synthetic Methods

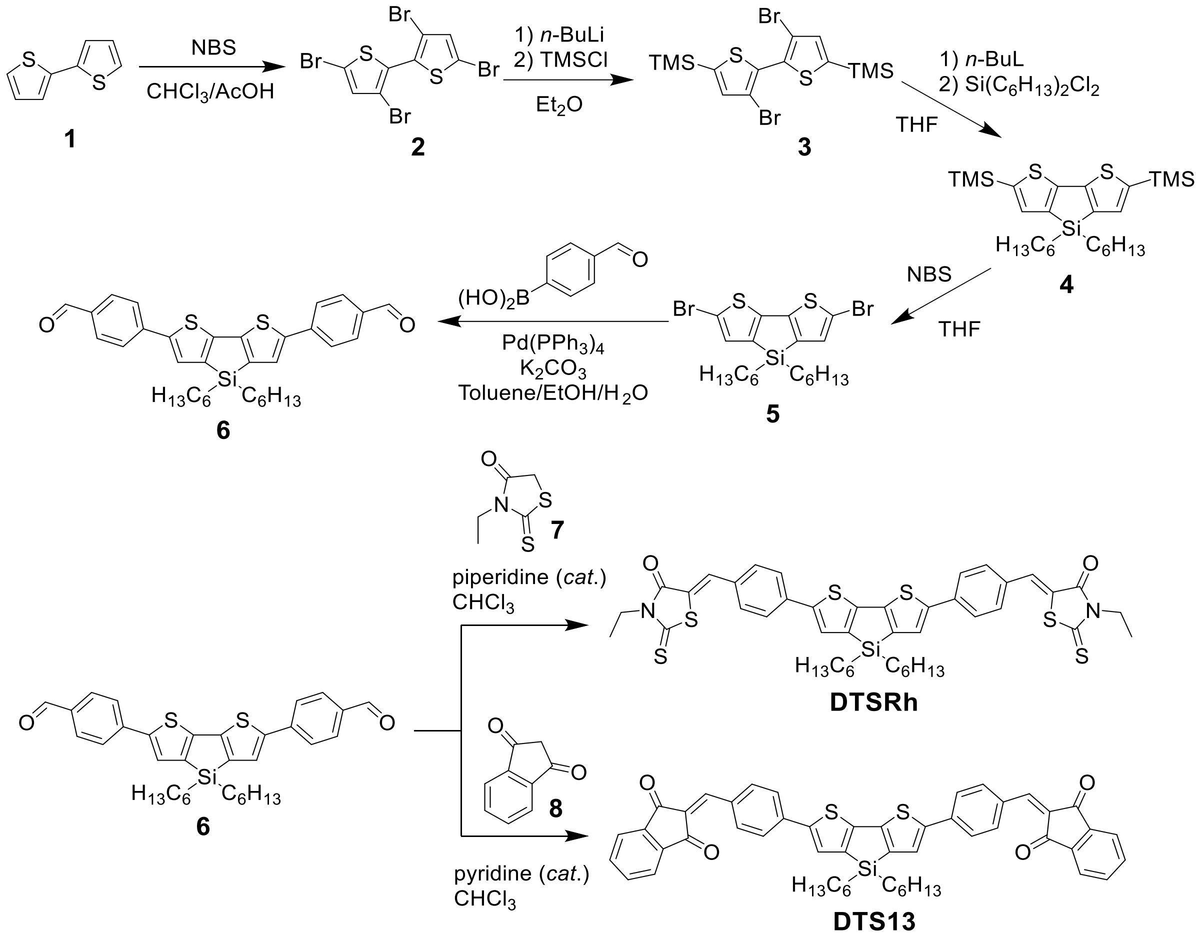

Scheme S1: Synthesis of **DTSRh** and **DTS13**. The dithieno[3,2-*b*:2′,3′-*d*]silole core was synthesised according to a modified a route of Oshita *et al.* [S10] which began with a fourfold bromination of 2,2'-bithiophene **1** to produce tetrabromide **2** in good yield. Protection of the terminal 5,5'-positions of **2** was achieved *via* lithium halogen exchange followed by addition of trimethylsilyl chloride to give **3** in moderate yield (68%). A second lithium halogen exchange, followed by addition of dichlorodi-(*n*-hexyl)silane provided the desired tercyclic core **4** (65%). Removal of the TMS groups and concomitant bromination of **4** was achieved using *N*-bromosuccinimide (NBS) in tetrahydrofuran (THF) in the absence of light to produce **5** in 68% yield. Compound **5** was then immediately subject to a Suzuki-Miyaura cross-coupling reaction with 4-formylbenzene boronic acid to afford the key intermediate dialdehyde **6** (60%).

Compound **6** was subject to Knoevenagel condensation with *N*-ethylrhodanine **7** to yield **DTSRh** in 76% yield while 1,3-indandione was introduced by using modified reactions conditions proposed by Zhang *et al.*[S11] yielding **DTS13** in 45%. Both compounds were purified using flash chromatography followed by slow reprecipitation in hexane at –18 °C.

3,3′,5,5′-Tetrabromo-2,2′-bithiophene (2)

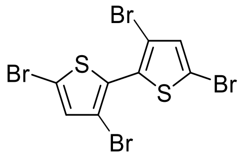

In a foil wrapped flask, *N*-bromosuccinimide (23.7 g, 130.7 mmol) was added portion wise to a solution of 2,2'-bithiophene (4.94 g, 29.7 mmol) in a mixture of $CHCl_3$ (40 mL) and glacial AcOH (40 mL) over a period of 3 hours. The brick red mixture obtained was left to stir overnight at room temperature. The solvent was removed under vacuum and the resulting solid was collected and washed first with a saturated $NaHCO_3$ solution (100 mL) and then *n*-hexane (100 mL) to yield a white powder. The crude product was recrystallised from boiling chloroform to yield **2** as an off-white crystalline solid (10.2 g, 71%). Mp. 140-142 °C. (Lit 139-140 °C).[S12]

Characterisation in agreement with literature.[S12]

^{1}H NMR (400 MHz, $CDCl_3$-*d*, ppm) δ = 7.05 (2H, s,).

^{13}C NMR (100 MHz, $CDCl_3$-*d*, ppm) δ = 133.1, 129.7, 115.0, 112.3.

3,3′-Dibromo-5,5′-bis(trimethylsilyl)-2,2′-bithiophene (3)

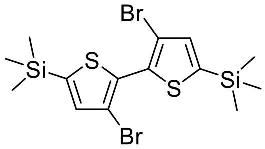

Compound **2** (5.00 g, 10.4 mmol) was dissolved in dry Et_2O (50 mL) and the resulting solution was cooled to −78 °C using an acetone/dry ice bath. *n*-BuLi (2.5 M in hexanes, 8.7 mL, 21.8 mmol) was added dropwise over 5 minutes and the reaction was left for 2 hours at −78 °C. Chlorotrimethylsilane (2.37 mL, 21.77 mmol) was then added dropwise, and the reaction was left to stir at −78 °C for a further 15 minutes before the reaction mixture was allowed to warm to room temperature and stirred overnight. The reaction mixture was then poured on to cold water (100 mL) and extracted into Et_2O (3 × 30 mL). The combined organic layers were washed with brine (50 mL) and dried over $MgSO_4$ before the removal of the solvent under reduced pressure to yield the crude product as a purple oil. Purification was achieved using flash column chromatography (SiO_2; 100% *n*-hexane; R_f = 0.49) to give **3** as a light yellow waxy solid (3.30 g, 68%). Mp. 86-87 °C. (Lit 87-88 °C).[S14]

The characterisation obtained was in good in agreement with literature.[S15]

^{1}H NMR (500 MHz, $CDCl_3$-*d*, ppm) δ = 7.15 (2H, s), 0.34 (18H, s).

^{13}C (100 MHz, $CDCl_3$-*d*, ppm) δ = 143.3, 137.4, 134.3, 113.3, 0.27.

4,4-dihexyl-2,6-bis(trimethylsilyl)-4*H*-silolo[3,2-*b*:4,5-*b*’] dithiophene (4)

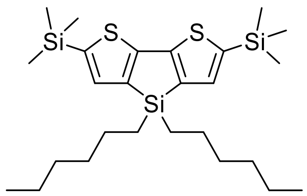

n-BuLi (1.90 mL, 2.5 M in hexanes 4.69 mmol) was added dropwise to a solution of **3** (1.00 g, 2.13 mmol) in anhydrous THF (20 mL) under argon at −78 °C *via* an acetone/dry ice bath. The resulting mixture was stirred for a further 30 minutes at this temperature before dichlorodihexylsilane (0.67 mL, 2.55 mmol) was added in one portion and the mixture was warmed to room temperature and left to stir 2 hours. After this period the mixture was quenched with water and extracted into Et_2O (3 × 20 mL). The combined organics were collected, dried over $MgSO_4$ and solvent was removed under reduced pressure. The crude solid was purified with flash column chromatography (SiO_2; 100% *n*-hexane; R_f = 0.77) to yield **4** as a colourless oil (0.74 g, 65%).

Characterisation in agreement with literature.[S15]

^{1}H NMR (400MHz, $CDCl_3$-*d*, ppm) δ = 7.12 (s, 2H), 1.45-1.35 (m, 4H), 1.33-1.21 (m, 16H), 0.90-0.84 (m, 6H), 0.33 (s, 18H).

^{13}C NMR (100 MHz, $CDCl_3$-*d*, ppm) δ = 154.6, 144.1, 141.2, 136.8, 33.0, 31.6, 24.4, 22.7, 14.2, 12.1, 0.3.

HRMS (ESI): m/z calcd for $C_{26}H_{46}S_2Si_3$: 506.2343 [M+]. Found: 506.2342.

2,6-dibromo-4,4-dihexyl-4*H*-silolo[3,2-*b*:4,5-*b*’] dithiophene (5)

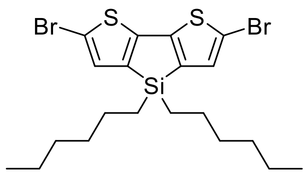

In a foil wrapped flask *N*-Bromosuccinimide (0.52 g, 2.91 mmol) was added to a solution of **4** (0.74 g, 1.46 mmol) in THF (40 mL) at 0 °C, the mixture was warmed to room temperate and stirred overnight. The resulting clear yellow solution was extracted into Et_2O (3 × 30 mL), dried over $MgSO_4$ and evaporated to dryness to yield a yellow residue which was purified using flash column chromatography (SiO_2; 100% *n*-hexane; R_f = 0.84) to yielding **5** as a yellow/green oil (0.61 g, 80%).

Characterisation in agreement with literature.[S15]

^{1}H NMR (400MHz, $CDCl_3$-*d*, ppm) δ = 7.00 (s, 2H), 1.35-1.22 (m, 16H), 0.89-0.84 (m, 10H).

^{13}C NMR (100 MHz, $CDCl_3$-*d*, ppm) δ = 149.1, 141.2, 132.3, 111.6, 32.9, 31.5, 24.1, 22.7, 14.2, 11.8.

HRMS (ESI): m/z calcd for $C_{20}H_{28}Br_2S_2Si$: 520.9763 [M+]. Found: 520.9761.

4,4'-(4,4-dihexyl-4*H*-silolo[3,2-*b*:4,5-*b*']dithiophene-2,6-diyl)dibenzaldehyde (6)

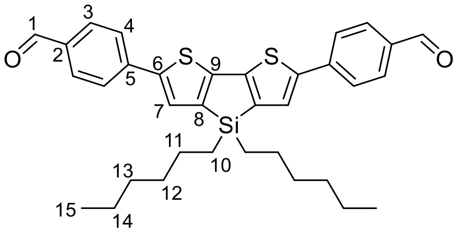

A solution of **5** (0.98 g, 1.88 mmol), 4-formylphenyboronic acid (0.62 g, 4.41 mmol), K_2CO_3 (1.55 g, 11.3 mmol) in a toluene: ethanol: H_2O (20 :3:3 mL) and degassed with argon for 1 hour. $Pd(PPh_3)_4$ (22 mg, 0.18 mmol)was added and the mixture was degassed for a further 15 minutes before being heated to 65 °C for 18 hours. The mixture was then cooled to room temperature and diluted with H_2O (30 mL). It was then extracted into CH_2Cl_2 (3 × 40 mL) and the organics where collected, dried over $MgSO_4$, and evaporated to dryness to yield a crude dark brown oil which was purified using flash column chromatography (SiO_2; 100% CH_2Cl_2; R_f = 0.36) to yield **6** as bright orange solid which was recrystalised in *n*-hexane: CH_2Cl_2 (0.60 g, 56 %). Mp. 114-115 °C.

^{1}H NMR (400MHz, $CDCl_3$-*d*, ppm) δ = 10.00 (s, 2H, C<u>H</u>O), 7.89 (d, 4H, Ar$\underline{H}^3$, *J* = 8.4. Hz), 7.76 (d, 2H, Ar$\underline{H}^4$, *J* = 8 Hz), 7.47 (s, 2H, Thio-<u>H</u>), 1.46-1.23 (m, 16H), 0.98 (q, 4H, -Si-C$\underline{H}_2$, *J* = 8, 7.6, 5.6 Hz), 0.85 (t, 6H, -C$\underline{H}_3$, *J* = 6.4, 7.2 Hz).

^{13}C NMR (400 MHz, $CDCl_3$-*d*, ppm) δ = 191.4 (C1), 150.2 (C9), 144.3 (C8), 140.4 (6), 134.9 (C2), 130.6 (C3), 128.0 (C7), 125.8 (C4), 125.6 (C5) ,32.9 (C13), 31.5 (31.5), 24.2 (C11/CC12), 22.6 (C11/C12), 14.1 (C15), 11.9 (C10).

Elemental analysis: Calculated for $C_{34}H_{38}O_2S_2Si$: 71.53 C%, 6.71 H%, 0.00 N%, Experimental 71.50 C%, 6.80 H%, 0.21 N%.

HRMS (ESI): *m/z* calcd for $C_{34}H_{38}O_2S_2Si$: 570.2077 [M+]. Found: 570.2076.

IR (v_{max}/cm^{-1}): 2918 (C-H), 2848 (C-H), 1692 (C=O), 1594 (C=C, Ar), 1560 (C=C, Ar), 1513 (C=C, Ar), 1308, 1210 (Si-C) ,1181, 1162, 1104, 1042.

(5*Z*,5'*Z*)-5,5'-(((4,4-dihexyl-4*H*-silolo[3,2-*b*:4,5-*b*']dithiophene-2,6-diyl)bis(4,1-phenylene))bis(methanylylidene))bis(3-ethyl-2-thioxothiazolidin-4-one) (DTSRh)

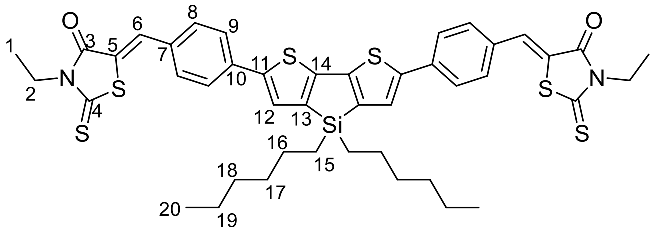

To a solution of dialdehyde (**6**) (0.10 g, 0.22 mmol) and *N*-ethylrhodanine (0.11 g, 0.65 mmol) in $CHCl_3$ (15 mL) in a foil wrapped round bottom flask, one drop of piperidine was added. The resulting solution was heated under reflux for 18 hours. After cooling to room temperature, the solvent volume was reduced to *circa* 5 mL and MeOH (20 mL) was added. The resulting precipitate was collected and purified using flash column chromatography (SiO_2; 100% CH_2Cl_2; R_f = 0.88) to yield a deep red solid which was further purified by dissolving in a minimum amount of CH_2Cl_2 and triturating in MeOH, then in *n*-hexane and cooling to −18 °C in a freezer. The resulting deep red precipitate was collected and dried under vacuum to yield **DTSRh** (40 mg, 21%). Mp: 215-216 °C.

^{1}H NMR (400MHz, $CDCl_3$-*d*, ppm) δ = 7.67 (4H, C=C<u>H</u>, 2H Ar-<u>H</u>, overlapping), 7.47 (4H, Ar-<u>H</u>, d, *J* = 8.7 Hz), 7.42 (2H, Thio-<u>H</u>, s), 4.20 (4H, N-C<u>H$_2$</u>, q, *J* = 7.1 Hz), 1.48-1.25 (22H, m), 0.98 (4H, Si-C<u>H$_2$</u>, t, *J* = 8.0 Hz), 0.86 (6H, CH_2-C<u>H$_3$</u>, t, *J* = 6.8 Hz).

^{13}C NMR (126 MHz, $CDCl_3$-*d*, ppm) δ = 192.9 (C4), 167.7 (C3), 149.9 (C14), 144.7 (C7), 144.4 (C13), 136.8 (C11), 132.4 (C8), 132.0 (C10), 131.6 (C9), 127.6 (C12), 126.1 (C6), 122.5 (C5), 40.0 (C2), 33.0 (C16/C17), 31.6 (C18/C19), 24.3 (C16/C17), 22.7 (C18/C19), 14.2 (C20), 12.4 (C1), 12.0 (C15).

Elemental analysis: Calculated for $C_{44}H_{48}N_2O_2S_6Si$: 61.64 C%, 5.64 H%, 3.27 N%, Experimental 61.61 C%, 5.32H%, 3.27 N%.

HRMS (ESI): *m/z* calcd for $C_{44}H_{48}N_2O_2S_6Si$: 856.1804 [M+]. Found: 856.1798.

IR (v_{max}/cm^{-1}): 2920 (C-H), 2851 (C-H), 1698(C=O), 1578 (C=C), 1547 (C=C, Ar), 1514 (C=C, Ar), 1381, 1346, 1327, 1244 (Si-C), 1223 (C-N), 1169 (C-N), 1133 (C=S), 1103 (C=S).

2,2'-(((4,4-dihexyl-4*H*-silolo[3,2-*b*:4,5-*b'*]dithiophene-2,6-diyl)bis(4,1-phenylene))bis(methanylylidene))bis(1*H*-indene-1,3(2*H*)-dione) (DTS13)

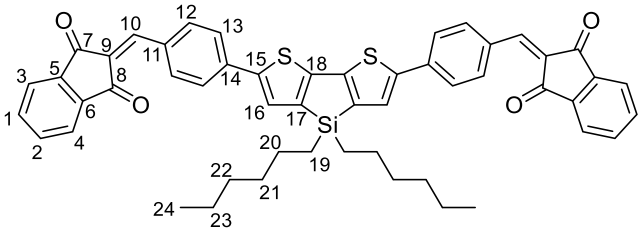

To a solution of dialdehyde (**6**) (0.10 g, 0.18 mmol,) and 1,3-indandione (0.10 g, 0.70 mmol) in $CHCl_3$ (5 mL) in a foil wrapped round bottom flask, one drop of pyridine was added. The resulting solution was heated under reflux for 18 hours. After cooling to room temperature, MeOH (50 mL) was added, and the resulting precipitate was collected and was purified using flash column chromatography (SiO_2; 100% $CHCl_3$; R_f = 0.50) to yield a deep purple solid which was further purified by dissolving in a minimum amount of CH_2Cl_2 then triturating in MeOH, then *n*-hexane and cooling to −18 °C in a freezer. The resulting deep purple was collected by filtration was washed with MeOH to yield **DTS13**. (65 mg, 45%). Mp. 227-228 °C.

^{1}H NMR (400MHz, $CDCl_3$-*d*, ppm) δ = 8.48 (4H, Ar-H, d, *J* = 8.7 Hz), 8.00-7.96 (4H, indan Ar-H, m), 7.80-7.76 (2H, C=CH, 4H indan Ar-H, m), 7.69 (4H, Ar-H,d, *J* = 8.7 Hz), 7.51 (2H, Thio-H, s), 1.49-1.42 (4H, -CH_2-CH_3 ,m) ,1.39-1.33 (4H, m), 1.28-1.26 (8H, m), 1.02-0.98 (4H, Si-CH_2, q, *J* = 8.0 Hz), 0.87 (6H, -CH_3, t, *J* = 6.8 Hz).

^{13}C NMR (126 MHz, $CDCl_3$-d, ppm) δ = 190.6 (C7/8), 189.3 (C7/8), 150.5 (C18), 146.0 (C15), 145.1 (C10), 144.6 (C17), 142.7 (C5/6), 140.2 (C5/6), 139.2 (C13), 135.4 (C12), 135.4 (C3/4), 135.2 (C3/4), 132.1 (C11), 128.4 (C9), 128.1 (C16), 125.4 (C13), 123.4 (C1/2), 123.3 (C1/2), 33.1 (-CH2-), 31.6 (-CH2-), 24.3(-CH2-), 22.7(-CH2-), 14.3 (C24), 12.0 (C19).

Elemental analysis: Calculated for $C_{52}H_{46}O_4S_2Si$: 75.51 C%, 5.61 H%, 0.00 N%, Experimental 75.14 C%, 5.20 H%, 0.12 N%.

HRMS (ESI): *m/z* calcd for $C_{52}H_{46}O_4S_2Si$: 826.2601 [M+]. Found: 826.2597.

IR (v_{max}/cm^{-1}): 2916 (C-H), 2849 (C-H), 1674 (C=O), 1561 (C=C), 1541 (C=C, Ar), 1511 (C=C, Ar), 1458 (C=C, Ar), 1422, 1345, 1319, 1250 (Si-C), 1213, 1184, 1151, 1019.

^{1}H and ^{13}C NMR Spectra

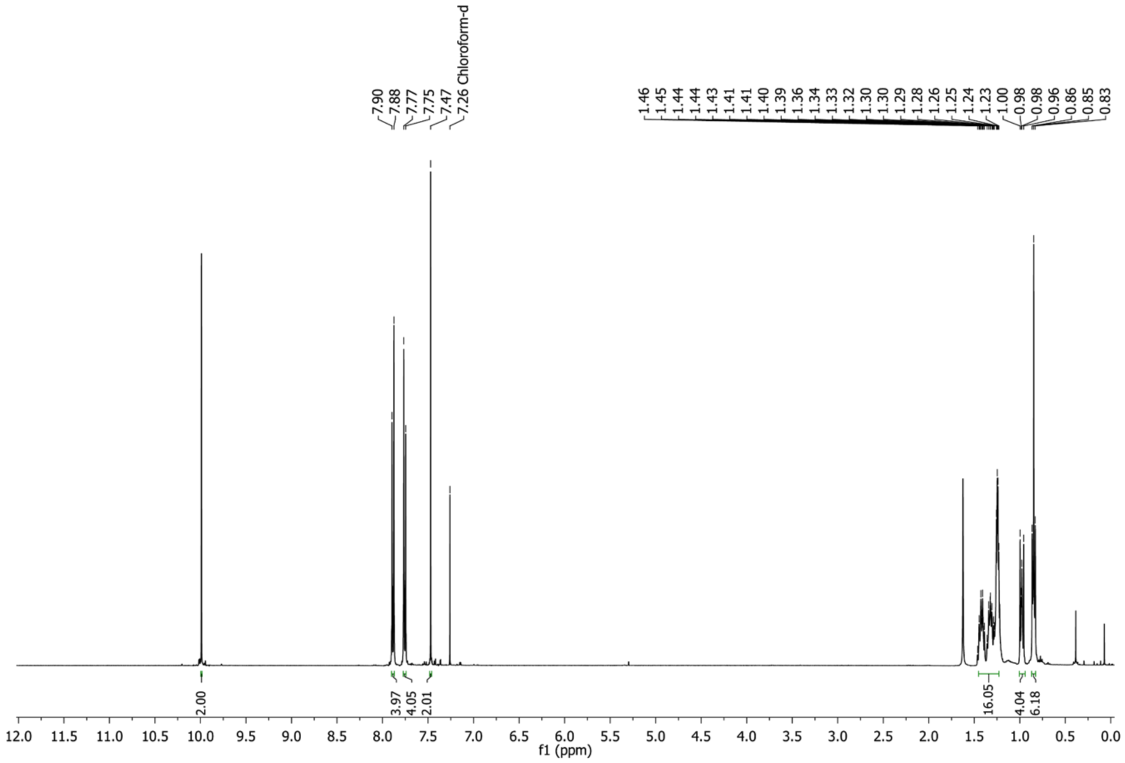

Spectrum S1: ^{1}H NMR spectrum for **6**.

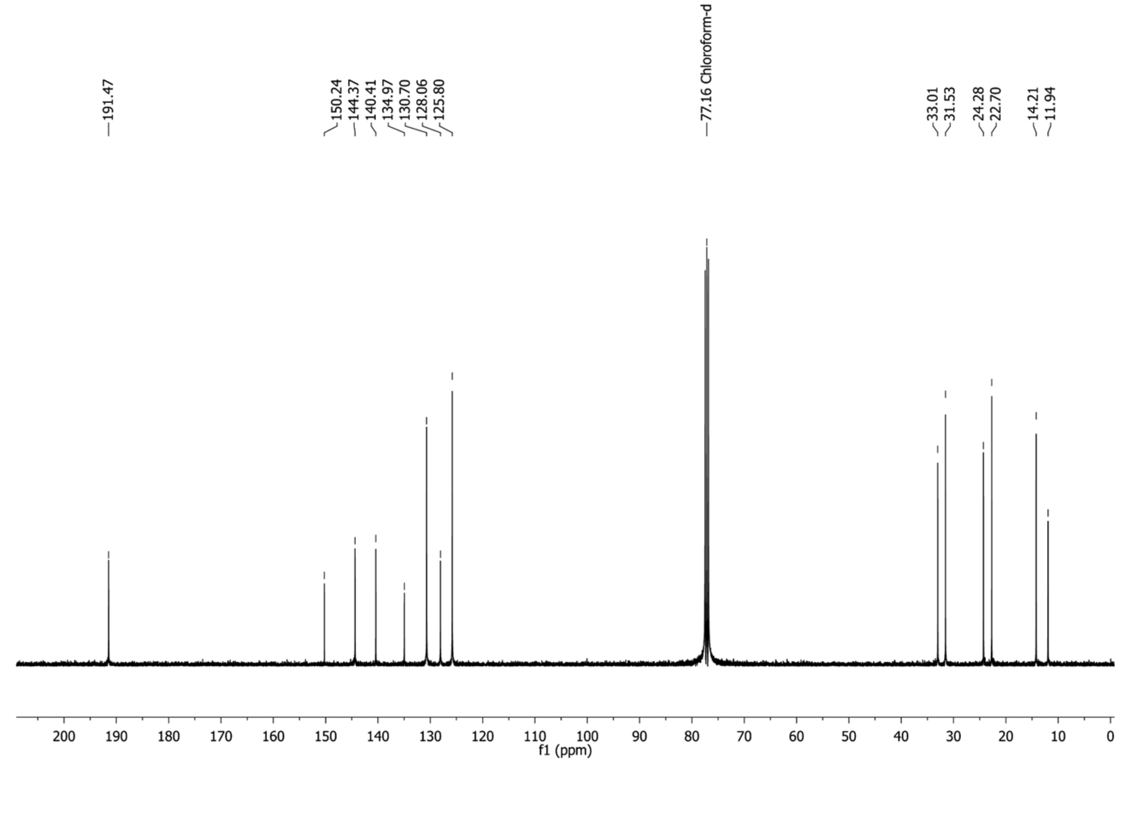

Spectrum S2: ^{13}C NMR spectrum for **6**.

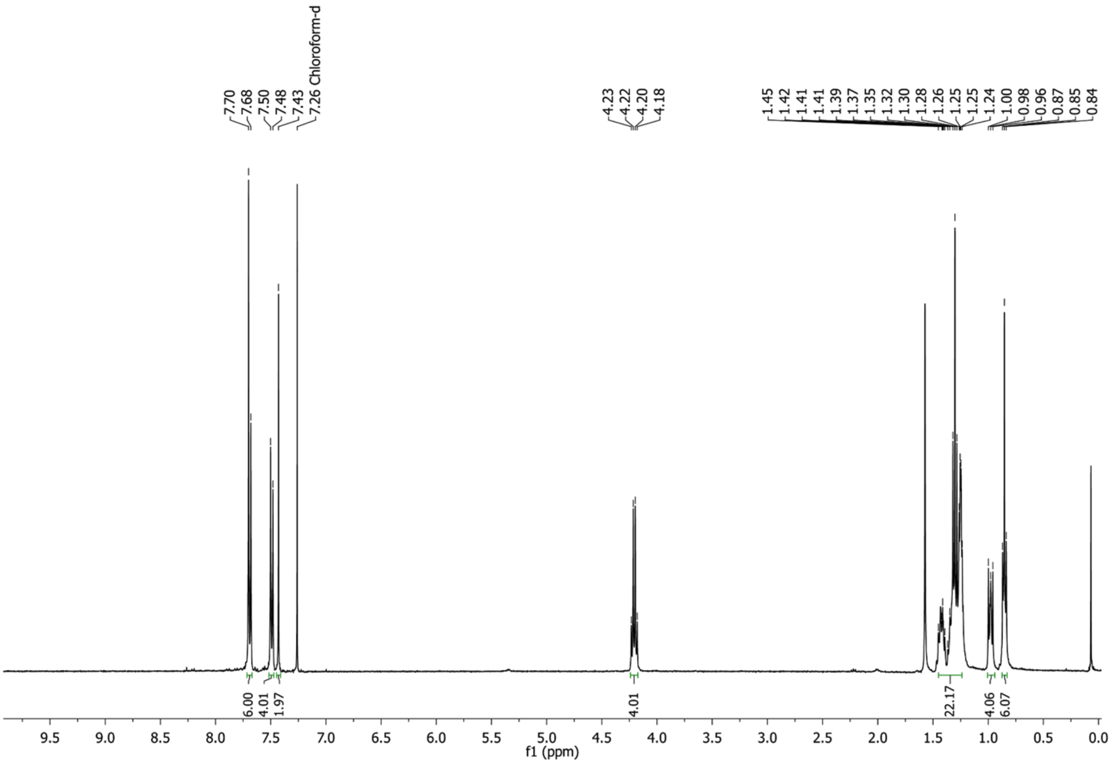

Spectrum S3: ^{1}H NMR spectrum for **DTSRh**.

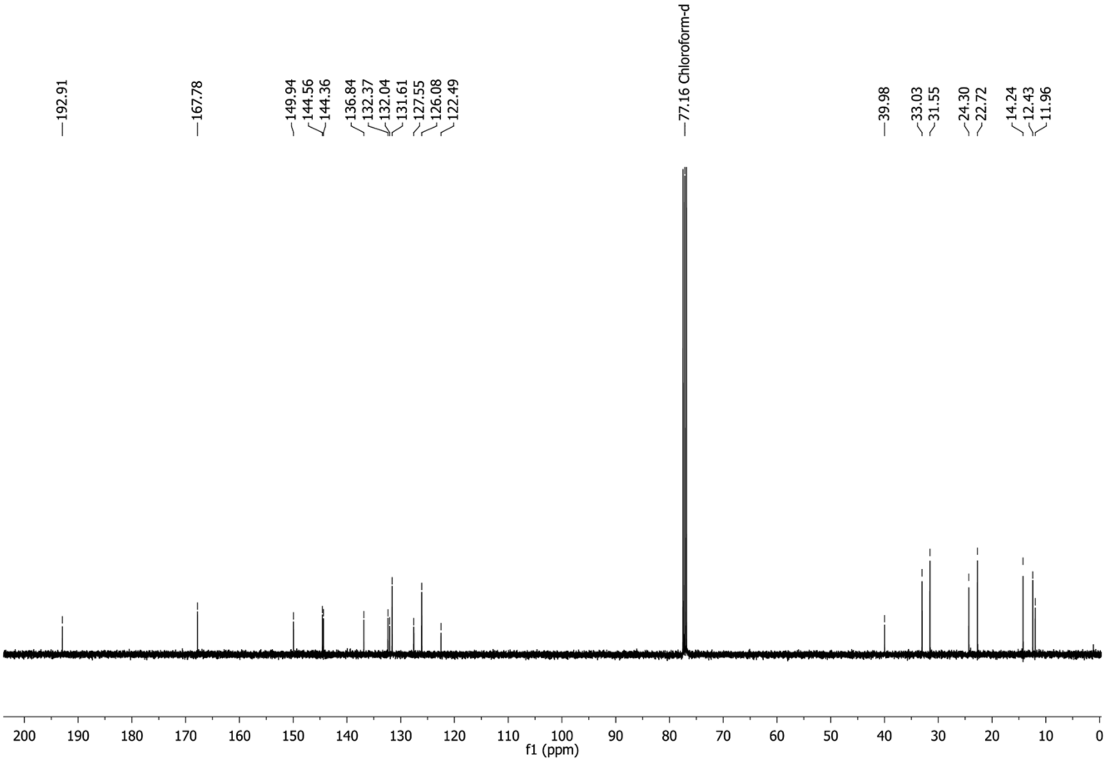

Spectrum S4: DTSRh ^{13}C NMR spectrum for **DTSRh**.

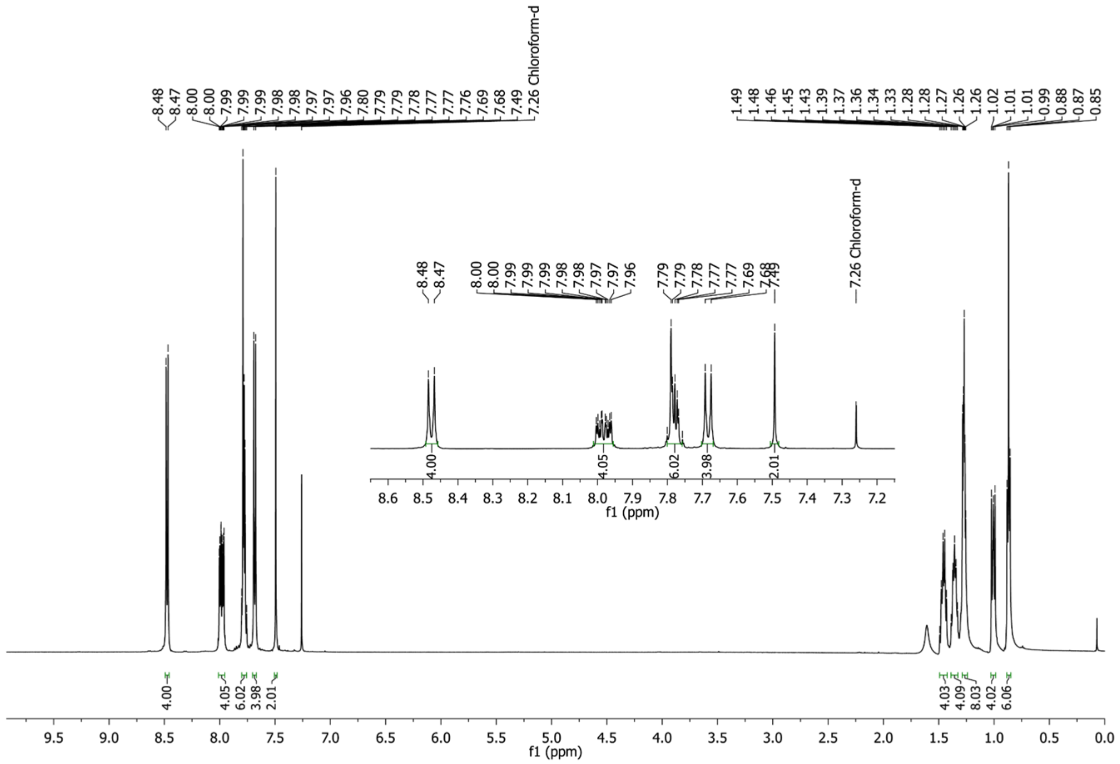

Spectrum S5: ^{1}H NMR spectrum for **DTS13**.

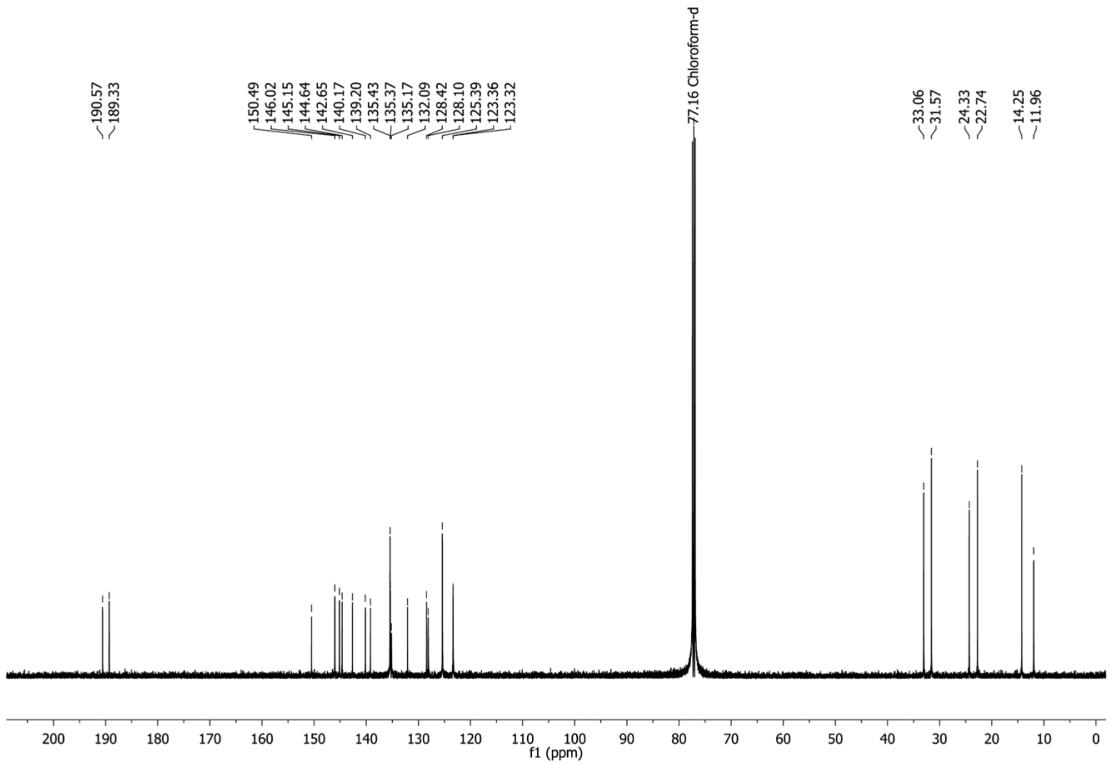

Spectrum S6: ^{1}H NMR spectrum for **DTS13**.

Molecular Weight and Optoelectronic Properties of PNF222

Table S1: Summary of molecular weight and the optical and electrochemical properties of polymer **PNF222** as purchased (Ossila, batch M2052A2).

M_w / M_n (PDI)[a]	IP (eV)	EA (eV)	λ_{max}^{film} (nm)	λ_{max}^{soln} (nm)	E_g^{opt} (eV)
163,267 / 81,850 (1.99)	−6.03[b] (−5.99)[c]	−4.14[b] (−3.90)[c]	695	681	1.68[d]

[a] Data from the Ossila website.[S16] [b] Experimentally measured in 0.1 M $TBAPF_6$ in acetonitrile with a conventional three-electrode configuration employing a platinum wire as a counter electrode, Ag/Ag^+ electrode as a reference electrode and drop cast films of **PNF222** on ITO as a working electrode at 0.1 Vs^{-1} [c] Brackets indicate literature values. Taken from Ref. 17 [d] Determined by the solution UV–vis absorption onset using $E_g^{opt} = 1240/\lambda_{onset}$

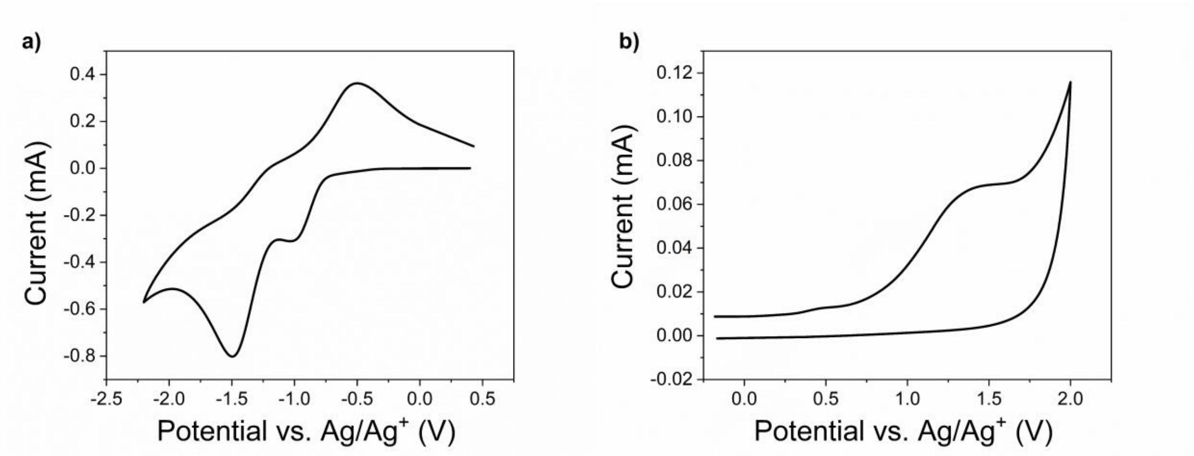

Figure S2: Cyclic voltammetry of **PNF222** showing a) reduction and b) oxidation.

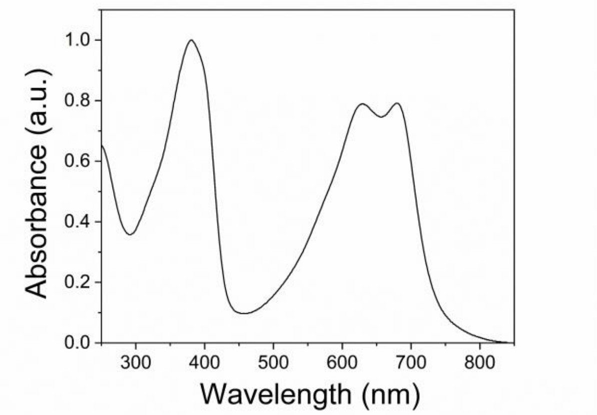

Figure S3: Solution state UV-vis spectra of **PNF222** in $CHCl_3$ at 10^{-5} M.

Optoelectronic Properties of DTSRh and DTS13

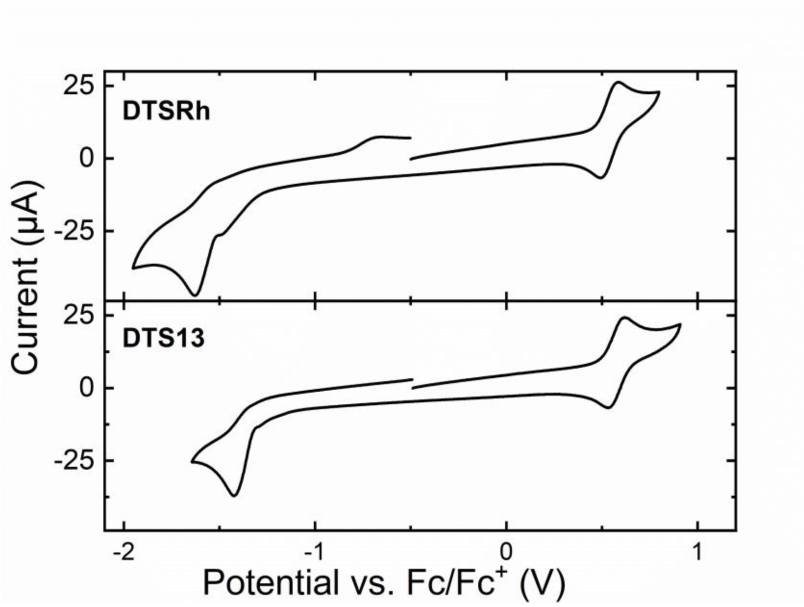

Figure S4 Cyclic voltammetry for **DTSRh** (upper) and **DTS13** (lower).

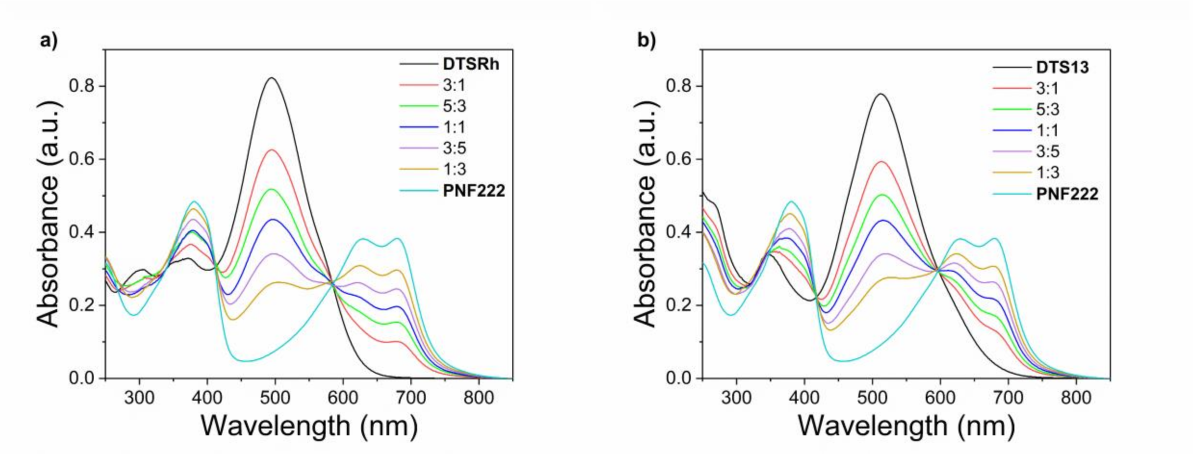

Figure S5 Solution phase UV/vis titrations of (a) **DTSRh** and (b) **DTS13** with **PNF222**

Table S2: UV/vis and cyclic voltammetry results for **DTSRh** and **DTS13**.

	λ_{max} (nm)	log ε	E_g^{opt} (eV)^a	E_g^{calc} (eV)^b	E^{ox} (V)^c	E^{rd} (V)^c
**DSTRh**	510	4.91	2.14	2.493	+0.54	−1.63^{irr}
**DTS13**	540	4.89	2.02	2.425	+0.57	−1.42^{irr}

$^a E_g^{opt} = 1240.68/\lambda_{onset}$. $^b E_g^{calc}$ obtained from DFT. ^{c}Reversible reductions are quoted as E_{half} otherwise peak potentials are provided. ^{irr} Irreversible peak

Computational Results

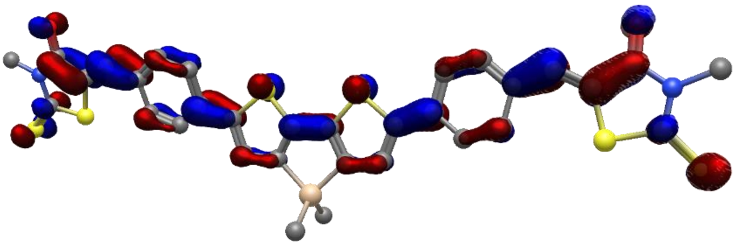

LUMO = −2.892 eV

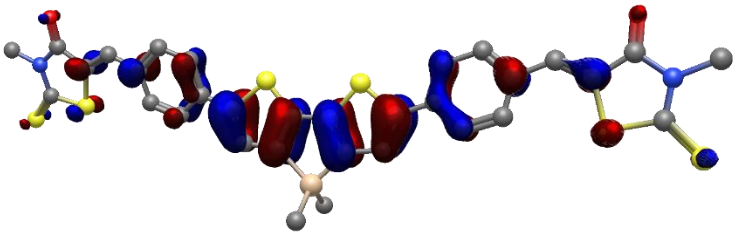

HOMO = −5.385 eV

Figure S6 DFT Optimised molecular structure and frontier orbital distributions for **DTSRh**. The ethyl chains on the terminal rhodanines and the *n*-hexyl chains of the central Si atom were replaced with methyl groups to reduce the computational cost.

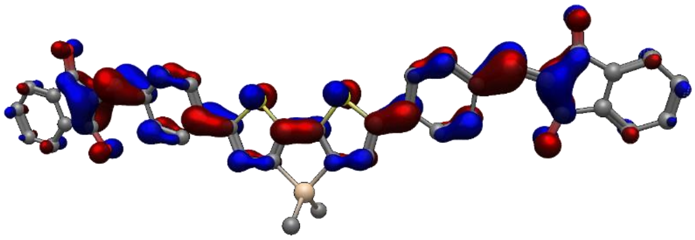

LUMO = −2.962 eV

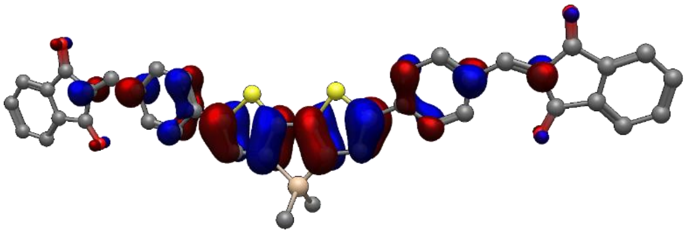

HOMO = −5.387 eV

Figure S7 DFT Optimised molecular structure and frontier orbital distributions for **DTS13**. The *n*-hexyl chains of the central Si atom were replaced with methyl groups to reduce the computational cost.

Cartesian Coordinates for DTSRh

C1	1.5306900000	-0.5266400000	-2.7676100000
Si2	2.7119100000	-1.2592600000	-1.4945400000
C3	2.1349400000	-2.9651500000	-0.9355100000
C4	4.5287000000	-1.2185900000	-2.0478700000
C5	5.3859000000	-1.7393900000	-3.0473400000
C6	6.7113400000	-1.3295200000	-2.9389500000
C7	7.8528900000	-1.6791400000	-3.7810200000
C8	7.7400500000	-2.6653800000	-4.7896000000
C9	8.8187500000	-3.0145800000	-5.5900800000
C10	10.0835000000	-2.4003100000	-5.4286600000
C11	11.2653700000	-2.7137200000	-6.2090800000
C12	10.1930100000	-1.4125000000	-4.4221200000
C13	9.1137600000	-1.0601100000	-3.6244000000
S14	6.9035800000	-0.2635700000	-1.5502400000
C15	5.2238700000	-0.3930800000	-1.1595700000
C16	4.4569400000	0.2049800000	-0.0859200000
C17	3.0914500000	-0.0920300000	-0.0445600000
C18	2.4567400000	0.5456800000	1.0490800000
C19	3.3077200000	1.3134500000	1.8375300000
C20	2.9982700000	2.0866200000	3.0379800000
C21	1.6611100000	2.3980700000	3.3794900000
C22	1.3531500000	3.1155900000	4.5273100000
C23	2.3666100000	3.5713000000	5.4035400000
C24	2.1350300000	4.3157400000	6.6267600000
C25	3.7030500000	3.2675700000	5.0536300000
C26	4.0114800000	2.5470200000	3.9086000000
S27	4.9474000000	1.2526000000	1.1999700000
H28	1.8650100000	0.4721400000	-3.0891800000
H29	1.4692000000	-1.1720000000	-3.6599900000
H30	1.1325200000	-2.9075800000	-0.4789200000
H31	2.8257600000	-3.3916200000	-0.1913600000
H32	5.0492000000	-2.4001700000	-3.8478400000
H33	6.7894900000	-3.1792800000	-4.9418700000
H34	8.6698000000	-3.7855000000	-6.3476700000
C35	11.4765800000	-3.6014700000	-7.2164200000

H36	11.1544400000	-0.9142400000	-4.2713400000
H37	9.2497300000	-0.2853000000	-2.8655500000
H38	1.3961700000	0.4359200000	1.2827200000
H39	0.8476900000	2.0847300000	2.7226100000
H40	0.3036300000	3.3268400000	4.7374500000
C41	0.9934900000	4.7591900000	7.2158600000
H42	4.5112100000	3.6028600000	5.7090600000
H43	5.0582600000	2.3197700000	3.6917600000
H44	0.5132500000	-0.4315000000	-2.3527100000
H45	2.0744000000	-3.6615900000	-1.7886600000
S46	-0.6890300000	4.6131100000	6.7067600000
C47	1.0681900000	5.5028500000	8.5036200000
C48	-1.2805900000	5.5022100000	8.1301300000
S49	-2.8716000000	5.8041900000	8.3998700000
N50	-0.2241200000	5.8736100000	8.9256200000
O51	2.0758500000	5.7660500000	9.1238500000
C52	-0.4139800000	6.6183500000	10.1627800000
H53	-1.0185300000	6.0320800000	10.8711100000
H54	-0.9436600000	7.5602700000	9.9580700000
H55	0.5806700000	6.8157000000	10.5806800000
C56	12.8293300000	-3.7185300000	-7.8275100000
S57	10.3705300000	-4.7372200000	-7.9908900000
H58	12.1662800000	-2.1447400000	-5.9519100000
C59	11.6376300000	-5.3561600000	-9.0750000000
N60	12.8235900000	-4.7071000000	-8.8318800000
C61	14.0391600000	-5.0197000000	-9.5707400000
S62	11.3681100000	-6.5348600000	-10.1856300000
H63	14.3207100000	-6.0710900000	-9.4087800000
H64	14.8261600000	-4.3502500000	-9.2026200000
H65	13.8757200000	-4.8711500000	-10.6482800000
O66	13.8124100000	-3.0762500000	-7.5265400000
H67	3.0331100000	4.5754200000	7.1989200000

Final single point energy = −4169.391419549399

Number of imaginary frequencies = 0

Cartesian Coordinates for DTS13

C1	-3.5532192960	-2.2780864727	2.2985173503
Si2	-4.1783847890	-0.9955275131	1.0662345644
C3	-4.4338125491	-1.7579846749	-0.6386956502
C4	-5.6853052310	-0.0190900328	1.6873744554
C5	-7.0407038285	-0.2038307865	2.0496915694
C6	-7.6923348173	0.9587945881	2.4522421354
C7	-9.0751008179	1.1293562402	2.8884638829
C8	-9.5411255649	2.3528073675	3.4231537629
C9	-10.8574439805	2.4981698867	3.8341002266
C10	-11.7878542781	1.4342360950	3.7379099079
C11	-13.1384102302	1.6890673445	4.1943575510
C12	-14.2860958706	0.9502886309	4.2519789253
C13	-14.6381818255	-0.4452803448	3.8613035215
C14	-16.0927260612	-0.6122969320	4.1856159752
C15	-16.8984918154	-1.7362492226	4.0039510155
C16	-18.2418197333	-1.6471489828	4.3867821547
C17	-18.7604030599	-0.4608753076	4.9371883920
C18	-17.9473622423	0.6641394622	5.1180525281
C19	-16.6098821566	0.5694458293	4.7344999851
C20	-15.5249116930	1.5947472475	4.8068693725
O21	-15.6283960204	2.7286872860	5.2315137780
O22	-13.9409045859	-1.3203832798	3.3752974000
C23	-11.3228922296	0.2072451560	3.2020768101
C24	-10.0057997191	0.0662682398	2.7908772582
S25	-6.5879682513	2.3290069509	2.3766404441
C26	-5.3000716570	1.3190947273	1.8183720630
C27	-3.9307012171	1.6412105164	1.4737210217
C28	-3.1259675816	0.5850849272	1.0347066566
C29	-1.8178231122	1.0263772541	0.7239023810
C30	-1.6096243858	2.3881063169	0.9214738439
C31	-0.3937622120	3.1669857162	0.7058144601
C32	-0.2463539169	4.4745795707	1.2227225021
C33	0.9180920501	5.1976645306	1.0124265212
C34	2.0026266817	4.6610466549	0.2757626827
C35	3.1749138597	5.4962191323	0.1164051088

C36	4.3744859415	5.3534894603	-0.5208826623
C37	4.9901612957	4.2694672103	-1.3400474003
C38	6.3369617970	4.7800007566	-1.7585000362
C39	7.3008433403	4.1464588592	-2.5429526652
C40	8.4923046949	4.8350689700	-2.7989038126
C41	8.7093089855	6.1245749789	-2.2799034794
C42	7.7390492445	6.7558711326	-1.4930270662
C43	6.5539503580	6.0652323305	-1.2421320227
C44	5.3591724568	6.4860042157	-0.4498387771
O45	5.2152005646	7.5403342849	0.1369525405
O46	4.5485089417	3.1740025786	-1.6446445395
C47	1.8553017494	3.3513170933	-0.2450473389
C48	0.6887479092	2.6314068134	-0.0332926955
S49	-3.0856727189	3.1500059260	1.5061490341
H50	-4.2744939448	-3.1057395325	2.4047463382
H51	-3.3971965434	-1.8292481094	3.2919800736
H52	-2.5952738967	-2.7089520146	1.9621699713
H53	-5.1757189640	-2.5730680606	-0.5974957576
H54	-3.4912360627	-2.1813134503	-1.0244226543
H55	-4.7913515619	-1.0052737949	-1.3586912166
H56	-7.5450214880	-1.1713134501	2.0336021356
H57	-8.8573828685	3.1991660866	3.5257960705
H58	-11.1866040108	3.4563110155	4.2455105895
H59	-13.2911155764	2.7053145766	4.5845801092
H60	-16.4807157013	-2.6500677645	3.5748057271
H61	-18.9013173648	-2.5096589144	4.2576370224
H62	-19.8141864679	-0.4214094976	5.2260897606
H63	-18.3351588780	1.5924505876	5.5442475096
H64	-12.0191845074	-0.6274780498	3.1114945034
H65	-9.6914661837	-0.8914548340	2.3720148045
H66	-1.0207837891	0.3662875694	0.3775173153
H67	-1.0519259000	4.9218980716	1.8103112024
H68	1.0056926571	6.2046591793	1.4294821664
H69	3.0963077239	6.4722307755	0.6159893423
H70	7.1174992906	3.1447746173	-2.9389798944
H71	9.2684694121	4.3676397113	-3.4108968874
H72	9.6502532254	6.6373638610	-2.4969728879

H73	7.8921023336	7.7568930841	-1.0826986868
H74	2.6714571401	2.9177893655	-0.8245183536
H75	0.6043195043	1.6332982398	-0.4672919396

Final single point energy = −2999.952583385568

Number of imaginary frequencies = 0

Dynamic Light Scattering Results

Table S3: Dynamic light scattering results for nanoparticles of **DTSRh**, **DTS13**, **PNF222** and combinations thereof.

	Z_{avg} (nm)	PDI
**DTSRh**	24.58	0.297
**DTS13**	33.27	0.181
**PNF222**	88.51	0.491
**DTSRh**:**PNF222** (3:1)	47.01	0.144
**DTSRh**:**PNF222** (1:1)	55.93	0.196
**DTSRh**:**PNF222** (1:3)	61.40	0.261
**DTSRh**:**PNF222** (3:1)	51.40	0.161
**DTSRh**:**PNF222** (1:1)	49.81	0.267
**DTSRh**:**PNF222** (1:3)	50.91	0.295

Table S4: Dynamic light scattering results for **DTS13**:**PNF222** (1:1) nanoparticles and also in the presence of either ascorbic acid (AA, black and red trace) or triethylamine (TEA, green and blue trace) before and after photoirradiation (solution degassed with N_2, 1.5 AG filter and 300 W Xe light source used for photoirradiation).

	Sacrificial oxidant	Z_{avg} (nm)	PDI
**DTS13**:**PNF222** (1:1)<br>Before photoirradiation	AA	91.35	0.392
**DTS13**:**PNF222** (1:1)<br>After photoirradiation	AA	71.88	0.291
**DTS13**:**PNF222** (1:1)<br>Before photoirradiation	TEA	68.55	0.390
**DTS13**:**PNF222** (1:1)<br>After photoirradiation	TEA	53.54	0.330

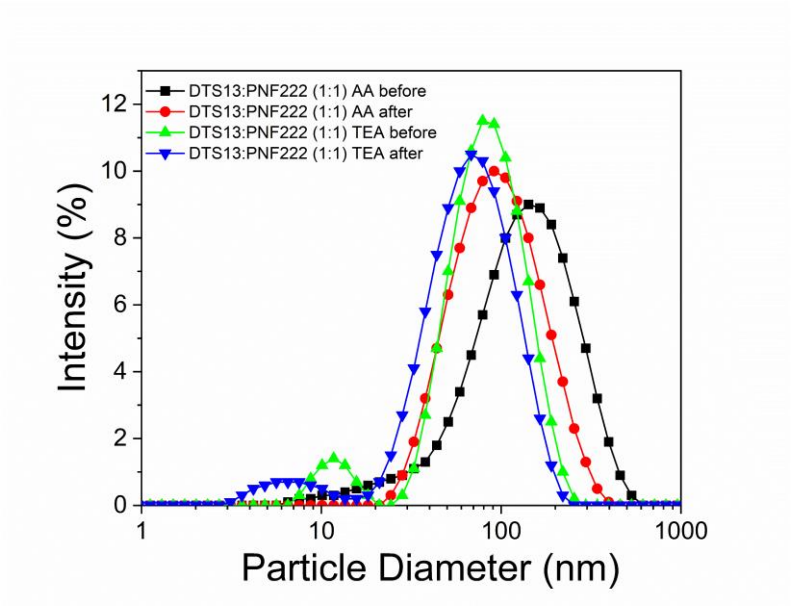

Figure S8: Dynamic light scattering results for **DTS13**:**PNF222** (1:1) nanoparticles in the presence of either ascorbic acid (AA, black and red trace) or triethylamine (TEA, green and blue trace) before and after photoirradiation (solution degassed with N_2, 1.5 AG filter and 300 W Xe light source used for photoirradiation).

Additional Photocatalysis Figures

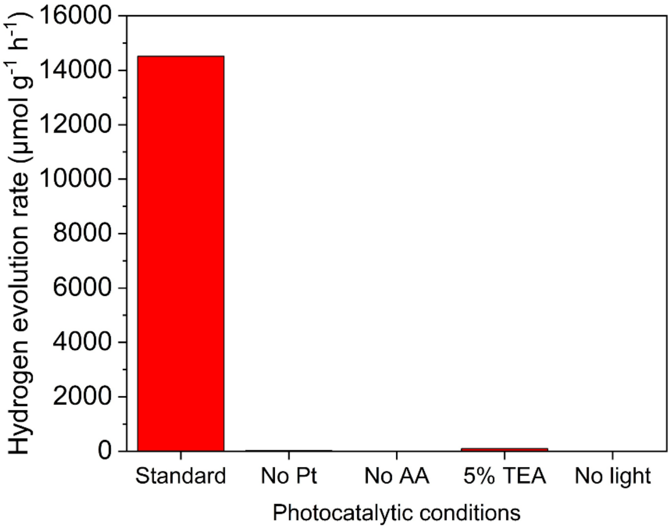

Figure S9: Control experiments relevant to the **DTS13**:**PNF222** (1:1) nanoparticles (as standard) including the absence of platinum cocatalyst, ascorbic acid (AA), triethylamine (TEA) used as an alternative sacrificial agent, and no light respectively. Experimental conditions: 2 mg nanoparticles from 0.5 mg/mL dispersions, the solution made up to a total volume up to 25 mL and degassing with N_2, atmospheric pressure, irradiated with a 300 W Xe light source equipped with an AM1.5G filter.

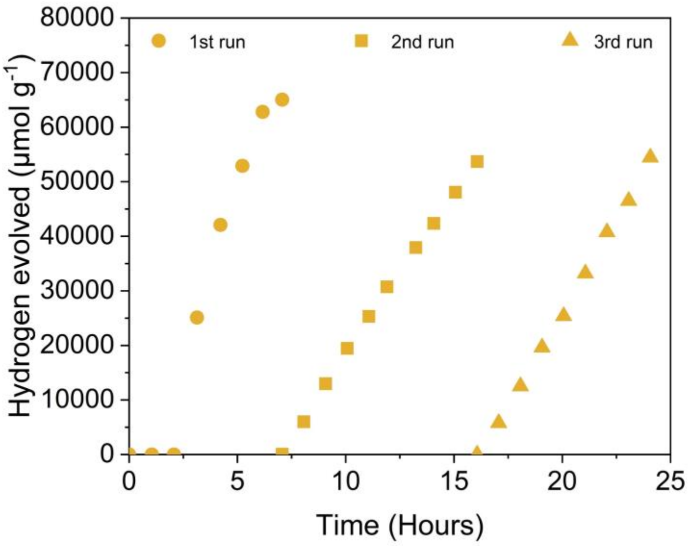

Figure S10: Mass normalised hydrogen evolution rate of **DTS13:PNF222** (1:1) ncBHJ nanoparticles over longer timescales. The first run started with two hours in the dark then the light source was initiated. After the first run, the photocatalytic reaction was degassed then the second run was commenced with the light source. Following the second run the photocatalytic reaction was degassed again and 5 mmol AA were added. Experimental conditions: 4 mL of a 0.5 mg mL^{-1} nanoparticle dispersion to obtain 2 mg of nanoparticles, 10 wt. % Pt (1.25 mL from a 0.4 mg mL^{-1} aqueous K_2PtCl_6 solution), 19.75 mL of a 0.2 M AA solution added to make the total volume up to 25 mL, degassed by nitrogen bubbling, irradiated with a 300 W Xe light source equipped with AM 1.5G filter).

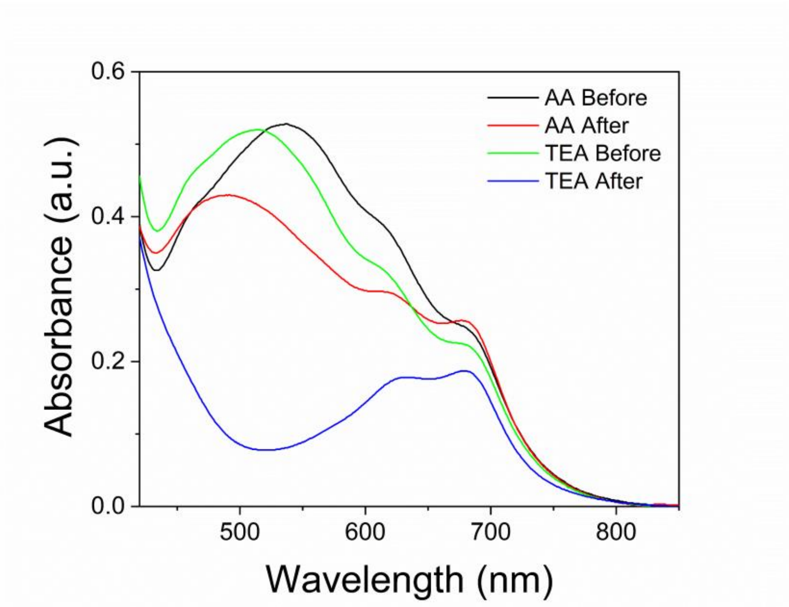

Figure S11: UV-vis spectroscopy of **DTS13**:**PNF222** (1:1) nanoparticles before and after photoirradiation (solution degassed with N_2, 300 W Xe light source equipped with an AM1.5G filter used for photoirradiation) using ascorbic acid (AA, black and red trace) and triethylamine (TEA, green and blue trace).

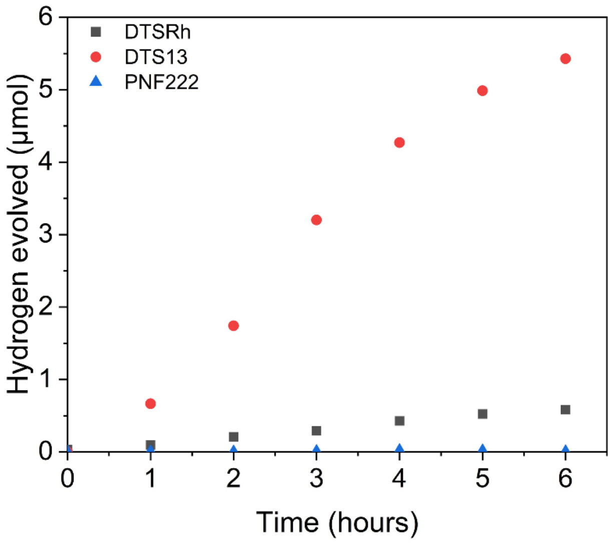

Figure S12: Photocatalytic hydrogen evolution experiment of the single component nanoparticles used in this study. Experimental conditions: 2 mg nanoparticles from 0.5 mg mL^{-1} dispersions, 10 wt% Pt loading (1.25 mL from a 0.4 mg mL^{-1} aqueous K_2PtCl_6 solution), 0.2 M ascorbic acid solution added to make the total volume up to 25 mL and degassing with N_2, atmospheric pressure, irradiated with a 300 W Xe light source equipped with an AM1.5G filter.

Table S5. Literature examples of photocatalytic hydrogen evolution performance of BHJ nanoparticles.

**Entry**	**BHJ components**	**Cocatalyst loading (wt% Pt)**	**Sacrificial reagent[a]**	**HER (μmol g^{-1} h^{-1})[b]**	**Surfactant**	**EQE / AQY**	**Ref.**
**1**	DTS13/PNF222	10	0.2 M AA pH 2	22,300	SDS	/	**This study**
**2**	PTB7-Th/EH-IDTBR	10	0.2 M AA pH 2	3,100[c]	SDS	/	13
**3**	PTB7-Th/EH-IDTBR	10	0.2 M AA pH 2	64,400[c]	TEBS	6.2% EQE at 700 nm	13
**4**	PCDTBT/$PC_{60}BM$	3	0.04 M AA	105,000	THF nanoprecipitation	3.72% AQY at 420 nm	S17
**5**	PFBT/PFODTBT/ITIC	6	0.8 M AA pH 4	60,800	PS-PEG-COOH	7% EQE at 600 nm	S18
**6**	PM6/ITCC-M	10	0.2 M AA pH 2.4	260,000[c]	SDBS	/	S19
**7**	PM6/ITCC-M/IDMIC-4F	10	0.2 M AA pH 2.4	306,000[c]	SDBS	5.9% AQY at 600 nm	S19
**8**	gIDTBT/oIDTBR	10	0.2 M AA pH 2	18,500[c]	TEBS	5.3% EQE at 400 nm	S20
**9**	PM6/Y6	10	0.2 M AA pH 2	43,900[c]	TEBS	5% EQE at 800 nm	S21
**10**	PM6/$PC_{71}BM$	5	0.2 M AA pH 2	73,700[c]	TEBS	8.7% EQE at 400 nm	S21
**11**	PM6/BTP-4F	15	0.2 M AA	64,300	TEBS	7.12% EQE at 800 nm	S22
**12**	PM6/BTP-FOH	15	0.2 M AA	78,400	TEBS	8.35% EQE at 800 nm	S22
**13**	PM6/BTP-2OH	15	0.2 M AA	102,100	TEBS	9.17% EQE at 800 nm	S22
**14**	PM6/TPP	20	0.2 M AA pH 2	72,700[c]	TEBS	8.55% EQE at 800 nm	S23
**15**	PM6/Y6CO	33	0.2 M AA	323,200[c]	TEBS	11.58% EQE at 700 nm	S24
**16**	PBDB-T/ITIC	10	0.2 M AA pH 3	257,000[c]	SDBS	9.9% AQY at 650 nm	S25
**17**	POZ-M/ITIC	6	0.2 M AA pH 4	63,000	PS-PEG-COOH	0.8% EQE at 450 nm	S26
**18**	PS/PBT	3	0.2 M AA	65,000[c]	THF nanoprecipitation	8.85% EQE at 405 nm	S27

[a] In some literature examples the pH is not explicitly stated but presumed to be unaltered which is pH 2 for a solution of ascorbic acid. [b] HER rounded to the nearest hundred. [c] Either reduced pressure is used or pressure is not explicitly stated in the photocatalytic reaction conditions.

Supplementary Information References

S1 S. C. Virgil, P. R. Jenkins, A. J. Wilson, M. D. García Romero, *N-Bromosuccinimide*. In *Encyclopedia of Reagent for Organic Synthesis,* Wiley, **2006**. https://doi.org/10.1002/047084289X.rb318.pub2

S2 A. M. Brouwer. Standards for photoluminescence quantum yield measurements in solution (IUPAC Technical Report). *Pure Appl. Chem.* **2011**, *83* (12), 2213.

S3 S. Trasatti. The Absolute Electrode Potential: An Explanatory Note. *Pure Appl. Chem.* **1986**, *58* (7), 955.

S4 N. G. Connelly, W. E. Geiger, Chemical Redox Agents for Organometallic Chemistry. *Chem. Rev.* **1996**, *96* (2), 877.

S5 A. J. Bard, L. R. Faulkner. *Electrochemical Methods: Fundamentals and Applications*, Wiley, New York, 2001.

S6 F. Neese. Software update: The ORCA program system—Version 5.0. *WIREs Comput. Mol. Sci.* **2022**, *12* (5), e1606.

S7 A. D. Becke. Density-functional thermochemistry. III. The role of exact exchange. *J. Chem. Phys.* **1993**, *98* (7), 5648.

S8 F. Weigend and R. Ahlrichs. Balanced basis sets of split valence, triple zeta valence and quadruple zeta valence quality for H to Rn: Design and assessment of accuracy. *Phys. Chem. Chem. Phys.* **2005**, *7* (18), 3297.

S9 F. Weigend. Accurate Coulomb-fitting basis sets for H to Rn. *Phys. Chem. Chem. Phys.* 2006, **8**, 1057.

S10 J. Ohshita, M. Nodono, T. Watanabe, Y. Ueno, A. Kunai, Y. Harima, K. Yamashita and M. Ishikawa. Synthesis and properties of dithienosiloles. *J. Organomet. Chem*. 1998, **553**, 487.

S11 Q. Zhang, Y. Sun, X. Chen, Z. Lin, X. Ke, X. Wang, T. He, S. Yin, Y. Chen and H. Qiu. An A_2–π–A_1–π–A_2-type small molecule donor for high-performance organic solar cells *J. Mater. Chem. C*, 2019, **7**, 5381.

S12 M. Takahashi, K. Masui, H. Sekiguchi, N. Kobayashi, A. Mori, M. Funahashi and N. Tamaoki. Palladium-Catalyzed C−H Homocoupling of Bromothiophene Derivatives and Synthetic Application to Well-Defined Oligothiophenes. *J. Am. Chem. Soc.* 2006, **128**, 10930.

S13 J.- H. Huang, C.- M. Teng, Y.- S. Hsiao, F.- W. Yen, P. Chen, F.- C. Chang and C.- W. Chu. Nanoscale Correlation between Exciton Dissociation and Carrier Transport in Silole-Containing Cyclopentadithiophene-Based Bulk Heterojunction Films. *J. Phys. Chem. C*, 2011, **115**, 2398.

S14 J. Ohshita, M. Nodono, H. Kai, T. Watanabe, A. Kunai, K. Komaguchi, M. Shiotani, A. Adachi, K. Okita, Y. Harima, K. Yamashita and M. Ishikawa. Synthesis and Optical, Electrochemical, and Electron-Transporting Properties of Silicon-Bridged Bithiophenes. *Organometallics*, 1999, **18**, 1453.

S15 A. A. El-Shehawy, N. I. Abdo, M. M. El-Hendawy, A.- R. I. A. Abdallah and J. S. Lee, *J. Phys. Org. Chem.* 2020, **33** (8), e4063.

S16 https://www.ossila.com/products/pnf222 Accessed (07/05/2024)

S17 H. Yang, X. Li, R. S. Sprick and A. I. Cooper, Conjugated polymer donor-molecular acceptor nanohybrids for photocatalytic hydrogen evolution. *Chem. Commun.* **2020**, *56* (50), 6790.

S18 A. Liu, L. Gedda, M. Axelsson, M. Pavliuk, K. Edwards, L. Hammarström, and H. Tian, Panchromatic Ternary Polymer Dots Involving Sub-Picosecond Energy and Charge Transfer for Efficient and Stable Photocatalytic Hydrogen Evolution. *J. Am. Chem. Soc.* **2021**, *143* (7), 2875.

S19 Y. Yang, D. Li, J. Cai, H. Wang, C. Guo, S. Wen, W. Li, T. Wang, D. Liu, Enhanced Photocatalytic Hydrogen Evolution from Organic Ternary Heterojunction Nanoparticles Featuring a Compact Alloy-Like Phase. *Adv. Funct. Mater.* **2023**, *33* (10), 2209643.

S20 J. Kosco, S. Gonzalez-Carrero, C. T. Howells, W. Zhang, M. Moser, R. Sheelamanthula, L. Zhao, B. Willner, T. C. Hidalgo, H. Faber, B. Purushothaman, M. Sachs, H. Cha, R. Sougrat, T. D. Anthopoulos, S. Inal, J. R. Durrant, I. McCulloch, Oligoethylene Glycol Side Chains Increase Charge Generation in Organic Semiconductor Nanoparticles for Enhanced Photocatalytic Hydrogen Evolution. *Adv. Mater.* **2022**, *34* (22), 2105007.

S21 J. Kosco, S. Gonzalez-Carrero, C. T. Howells, T. Fei, Y. Dong, R. Sougrat, G. T. Harrison, Y. Firdaus, R. Sheelamanthula, B. Purushothaman, F. Moruzzi, W. Xu, L. Zhao, A. Basu, S. De Wolf, T. D. Anthopolous, J. R. Durrant, I. McCulloch, Generation of long-lived charges in organic semiconductor heterojunction nanoparticles for efficient photocatalytic hydrogen evolution. *Nat. Energy*, **2022**, *7* (4), 340.

S22 X. Liu, Y. Zhao, Y. Ni, F. Shi, X. Guo, C. Li. Hydroxylated organic semiconductors for efficient photovoltaics and photocatalytic hydrogen evolution. *Energy Environ. Sci.* **2023**, *16* (9), 4065.

S23 Z. Zhang, W. Si, B. Wu, W. Wang, Y. Li, W. Ma, Y. Lin. Two-Dimensional Polycyclic Photovoltaic Molecule with Low Trap Density for High-Performance Photocatalytic Hydrogen Evolution. *Angew. Chem. Int. Ed.* **2022**, *61* (10), e202114234

S24 Y. Liang, T. Li, Y. Lee, Z. Zhang, Y. Li, W. Si, Z. Liu, C. Zhang, Y. Qiao, S. Bai, Y. Lin. Organic Photovoltaic Catalyst with σ-π Anchor for High-Performance Solar Hydrogen Evolution, *Angew. Chem. Int. Ed.* **2023**, *62* (12), 202217989.

S25 Y. Yang, D. Li, P. Wang, X. Zhang, H. Zhang, B. Du, C. Guo, T. Wang, D. Liu. Polymer/non-fullerene acceptor bulk heterojunction nanoparticles for efficient photocatalytic hydrogen production from water. *Polymer*, **2022**, *244*, 124667.

S26 M. V. Pavliuk, S. Wrede and H. Tian. Phenoxazine-based small molecule heterojunction nanoparticles for photocatalytic hydrogen production. *Chem. Commun.*, **2023**, *59* (37), 5611.

S27 M. Yu, W. Zhang, X. Liu, G. Zhao, J. Du, Y. Wi, W.-H. Zhu. Energy transfer enhanced photocatalytic hydrogen evolution in organic heterostructure nanoparticles via flash nanoprecipitation processing. *Green Energy Environ*., **2024**, advance article.
